# Dual‐Substrate Synergistic Photocatalysis: Exogenous Reagent‐Free Co‐Removal of Phenol and Cr(VI) via Electron‐Donor‐Mediated Redox Coupling over Modified Carbon Nitride

**DOI:** 10.1002/advs.75763

**Published:** 2026-06-01

**Authors:** Xiaoman Zhang, Qiyu Wang, Xiaodan Liu, Shuqi Wan, Cuiwei Du, Qilu Li, Chongfei Yu, Shuying Dong, Xianfa Su, Chun Hu

**Affiliations:** ^1^ School of Environment Key Laboratory for Yellow River and Huai River Water Environmental and Pollution Control Henan Normal University Ministry of Education Henan Key Laboratory for Environmental Pollution Control Xinxiang Henan P. R. China; ^2^ Institute of Environmental Research at Greater Bay Key Laboratory for Water Quality and Conservation of the Pearl River Delta Ministry of Education Guangzhou University Guangzhou P. R. China

**Keywords:** Cr(VI) reduction, dual‐substrate system, electron donor mechanism, phenol degradation, synergistic photocatalysis

## Abstract

Traditional photocatalytic systems for pollutant removal depend on exogenous oxidants or sacrificial agents, hampering sustainability. Herein, we report an exogenous reagent‐free dual‐substrate cooperative strategy. Without adding external chemicals (e.g., methanol, sulfite), the target pollutant phenol is directly used as an endogenous electron donor to drive the reduction of Cr(VI) and its own degradation over a thermally exfoliated graphitic carbon nitride (CN‐T) catalyst. Phenol fulfills two critical roles in this system: (i) it is oxidized and degraded by photogenerated holes (h^+^), and (ii) it transfers electrons to CN‐T, enhancing Cr(VI) reduction by photogenerated electrons (e^−^). Kinetic studies, DFT calculations, and EPR spectroscopy reveal that phenol adsorbs on CN‐T via π‐π stacking and hydrogen bonding, facilitating electron transfer from its HOMO to the CN‐T LUMO. This process suppresses e^−^‐h^+^ recombination and promotes Cr(VI) reduction. In the coexisting system, CN‐T achieved 76.4% Cr(VI) reduction and 99.8% phenol degradation, with a 6.4‐fold higher Cr(VI) reduction rate constant than in the single pollutant system. Substituted phenols (4‐methylphenol, 4‐chlorophenol, p‐nitrophenol) strongly correlate with electronic descriptors (Hammett constant, E_HOMO_), establishing a robust quantitative structure‐activity relationship (QSAR) for predictive synergistic co‐removal. This work advances photocatalytic redox coupling and provides a “waste‐to‐waste” strategy for sustainable complex wastewater.

## Graphical abstract

An exogenous reagent‐free dual‐substrate strategy harnesses phenol as an intrinsic electron donor to drive synchronous phenol degradation and Cr(VI) reduction over modified carbon nitride. π–π stacking and hydrogen bonding direct electron transfer to the catalyst, enriching photogenerated electrons and accelerating Cr(VI) reduction 6.4‐fold. This waste‐to‐waste approach enables sustainable remediation of complex coexisting wastewater.

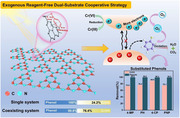

## 1 Introduction

Rapid industrialization has led to the simultaneous discharge of organic pollutants and heavy metals into aquatic ecosystems, creating complex contamination matrices that challenge conventional remediation technologies [1]. Phenolic compounds, ubiquitous in effluents from coal chemical, petroleum refining, and pharmaceutical industries, exhibit acute toxicity and recalcitrance to biodegradation [2, 3]. Concurrently, Cr(VI), a mobile heavy metal from electroplating and leather tanning processes, poses severe carcinogenic risks and persists in the environment [4, 5]. The co‐occurrence of these pollutants in industrial wastewater necessitates innovative strategies capable of synchronous removal, as sequential treatment approaches often suffer from process inefficiencies and secondary pollution [6, 7].

Photocatalysis has emerged as a promising green technology for water purification, leveraging solar energy to drive redox reactions without hazardous byproducts [8]. However, conventional photocatalytic systems face critical limitations: single‐substrate reactions underutilize photogenerated charge carriers (electrons/holes), while sacrificial‐agent‐aided processes rely on exogenous chemicals (e.g., methanol, sulfites) that increase operational costs and introduce secondary contaminants [9, 10, 11]. In addition, many reported co‐removal systems for mixed pollutants depend on external oxidants (e.g., O_3_ or peroxide‐based processes) to drive redox reactions, further increasing energy and chemical input [12]. This study addresses these drawbacks by proposing an exogenous oxidant/sacrificial agent‐free dual‐substrate synergistic mechanism where target pollutants themselves act as electron donors/acceptors, enabling concurrent remediation without external additives [13, 14, 15]. Notably, by utilizing intrinsic electron donor behavior rather than external reagents, such a strategy is expected to reduce operational costs; previous studies have reported that eliminating reagent input can lower treatment costs by approximately 8–10% [16]. This approach, therefore, represents a more sustainable and economically favorable pathway, aligning with the global demand for green environmental technologies.

Graphitic carbon nitride (g‐C_3_N_4_) is a compelling photocatalyst due to its visible‐light responsiveness, chemical stability, and low cost [17, 18, 19]. Yet, its practical application is hindered by rapid electron‐hole recombination and inefficient carrier utilization in single‐pollutant systems [20, 21]. While bifunctional photocatalytic systems have shown potential—such as simultaneous organic oxidation and H_2_ production—they predominantly focus on resource recovery rather than multi‐pollutant remediation [22, 23]. Critical knowledge gaps remain: (i) The molecular mechanisms underlying synergistic interactions between co‐existing pollutants in photocatalytic systems; (ii) The influence of pollutant‐specific properties (e.g., substituent electronic effects) on redox coupling efficiency; (iii) The role of solution chemistry (e.g., pH) in modulating charge transfer dynamics between substrates and catalysts.

This work presents a paradigm shift by engineering a CN‐T photocatalytic system—prepared via thermal exfoliation of pristine carbon nitride (CN) as detailed in our recent publication [24]—that harnesses phenol as an intrinsic electron donor to facilitate concurrent phenol degradation (by h^+^) and Cr(VI) reduction (by e^−^) without external reagents, creating a self‐sustaining redox cycle. By integrating kinetic analysis, density functional theory (DFT) calculations, and electron paramagnetic resonance (EPR) spectroscopy, we elucidate a dual‐adsorption‐mediated electron transfer pathway. Specifically, phenol binds to CN‐T via π‐π stacking and hydrogen bonding, promoting directed electron flow to Cr(VI). This mechanism not only suppresses charge recombination but also enables “waste‐to‐waste” remediation by utilizing pollutant‐pollutant interactions. Furthermore, we validate the system's generality with substituted phenols and establish quantitative structure‐activity relationships. This research advances the understanding of photocatalytic redox coupling and provides a scalable solution for complex wastewater treatment.

## 2 Experimental Section

### 2.1 Catalyst Synthesis and Characterization

The information regarding chemicals and reagents used in this study is presented in Text S1. CN (pristine g‐C_3_N_4_) and CN‐T were synthesized following our previous research protocols. Detailed synthesis procedures and material characterizations are provided in the Supporting Information (Text S2).

### 2.2 Photocatalytic Performance Test

#### 2.2.1 Single‐Pollutant Systems

For phenol degradation, 50 mg CN‐T was dispersed in 100 mL of 10 mg/L phenol solution, and the suspension was continuously stirred at a constant rate of 500 rpm throughout the entire reaction process, including 30 min of dark equilibration and subsequent visible‐light irradiation with a 30 W LED source (λ ≥ 420 nm). Aliquots (4 mL) were filtered (0.22 µm) and analyzed by high‐performance liquid chromatography (HPLC).

For the reduction of Cr(VI), 50 mg CN‐T was added to 100 mL of 5 mg/L Cr(VI) solution. Following dark adsorption, the suspension was irradiated, and Cr(VI) concentrations were measured using the diphenylcarbazide (DPC) colorimetry via UV–vis spectroscopy (λ = 540 nm) after filtration.

#### 2.2.2 Coexistence System

The procedure was identical to the single‐pollutant systems, but using a mixed solution of 10 mg/L phenol and 5 mg/L Cr(VI). Phenol and Cr(VI) concentrations were analyzed as described above.

### 2.3 Mechanistic Investigations

#### 2.3.1 Active Species Identification

To identify the reactive species generated during photocatalysis, specific scavengers were introduced into the reaction mixture. Generally, isopropanol (IPA), nitrogen (N_2_) condition, ammonium oxalate (AO), and potassium bromate (KBrO_3_) were added into the reaction system to detect the •OH, •O_2_^−^, h^+^ and e^−^, respectively. In Electron paramagnetic resonance (EPR) experiments, 5,5‐dimethyl‐1‐pyrroline‐N‐oxide (DMPO) was used to detect •O_2_^−^, and 2,2,6,6‐tetramethyl‐1‐piperidinyloxy (TEMPO) was applied to detect h^+^. Besides, the relative inhibition rate (R) was calculated to evaluate the specific contribution of each active species, which refers to the decrease in the degradation rate constant in the presence of a single quencher. The relative inhibition rate of free radicals (h^+^, •O_2_^−^, •OH) are calculated based on the following equations:

(1) $$R\;\left(h^{+}\right)=\frac{k\left(h^{+}\right)}{k_{0}}\;\approx \frac{k_{0}-k\left(\mathrm{AO}\right)}{k_{0}}$$

(2) $$R\;\left(•O_{2}^{-}\right)=\frac{k\left(•O_{2}^{-}\right)}{k_{0}}\;\approx \frac{k_{0}-k\left(N_{2}\right)}{k_{0}}$$

(3) $$R\;\left(•\mathrm{OH}\right)=\frac{k\left(h^{+}\right)}{k_{0}}\;\approx \frac{k_{0}-k\left(\mathrm{IPA}\right)}{k_{0}}$$

Notably, k_0_ represents the apparent rate constant of phenol. Also, k_IPA_, k_N2_, and k_AO_ are the apparent rate constants with the corresponding radical scavenger in the phenol degradation process, which reflect the regulatory weight of the species on the reaction (not the absolute proportion, due to the synergistic effect between species) [25].

#### 2.3.2 Electron Transfer and Interfacial Characterization

Density functional theory (DFT) calculations (Gaussian 16) explored adsorption configurations and frontier molecular orbitals. HOMO‐LUMO values of the pollutants, electrostatic potential calculations were computed and plotted by Multiwfn in combination with the VMD program [26, 27, 28, 29]. Transient photocurrent measurements analyzed charge separation efficiency. In addition, Fourier transform infrared (FTIR) spectroscopy was used to track phenol adsorption and degradation intermediates on the catalyst surface. The details of the theoretical calculation settings and procedures are provided in the Supporting Information (Text S3).

## 3 Results and Discussion

### 3.1 Synergistic Co‐Removal Performance in Single vs. Coexisting Systems

**FIGURE 1:**
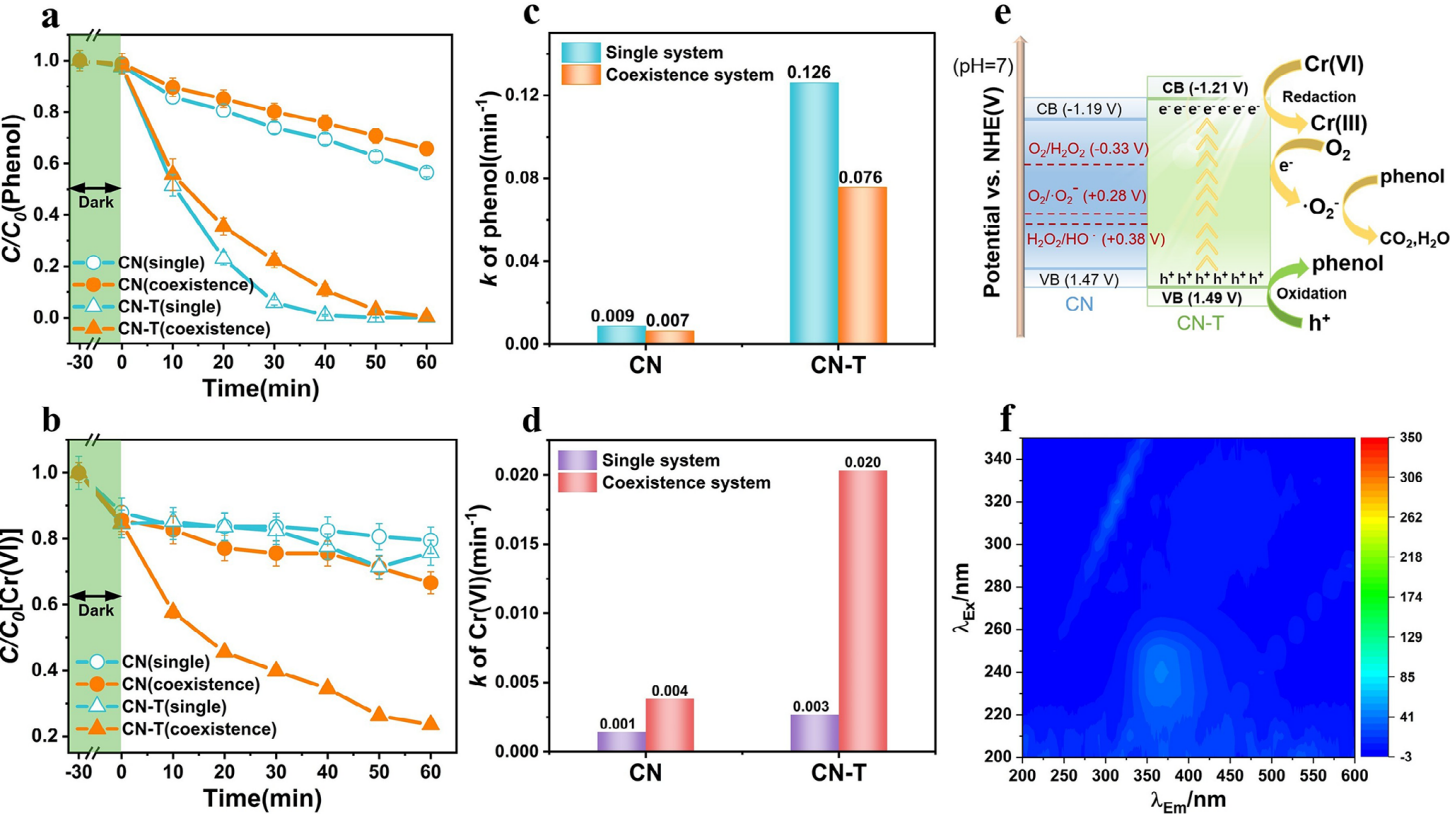
(a) Phenol degradation and (c) associated rate constant (k) for CN and CN‐T in single and coexistence systems. (b) Cr(VI) reduction and (d) associated rate constant (k). (e) Determined energy band alignments and proposed reaction pathway mechanism for CN‐T. (f) 3D EEM of phenol after 60 min degradation in the coexistence system.

Control experiments performed in the absence of a catalyst, under both single and mixed pollutant conditions, were carried out to verify that the elimination of phenol and Cr(VI) was primarily driven by photocatalysis. Negligible removal of both pollutants was observed (Figure S1), confirming their chemical stability under visible light. Systematic optimization of catalyst dosage (0.25–0.75 g/L) identified 0.5 g/L as optimal (Figure S2), which was adopted in all subsequent experiments. Figure 1a–d illustrates the photocatalytic degradation behavior in single and coexisting systems. In the single phenol system, CN removed only 34.3% of phenol within 60 min, whereas CN‐T achieved complete degradation with a removal rate approximately three times that of CN. The first‐order kinetic constants (k) fitting results further verified the superior catalytic performance of CN‐T, with a degradation rate constant of CN‐T (k = 0.126 min^−1^, R^2^ = 0.98) being approximately 14 times higher than that of CN (k = 0.009 min^−1^, R^2^ = 0.99) (Figure 1c). In the coexistence system, the CN catalyst exhibited a similar removal efficiency of phenol to that observed in the single system. Although the degradation rate of CN‐T slightly decreased in the coexistence system, it still maintained excellent catalytic stability, with a phenol removal rate of approximately 99.0% within 60 min.

For Cr(VI) reduction, both CN and CN‐T exhibited minimal activity within the single Cr(VI) system (Figure 1b). Within 60 min of being subjected to the coexistence system, the extent of Cr(VI) reduction reached 33.4% for CN, compared to 76.4% for CN‐T. Kinetic constant analysis (Figure 1d) indicates that, in the coexisting system, the reduction rate constant of CN (k = 0.004 min^−1^) was 2.7 times higher than in the single system (k = 0.001 min^−1^), while for CN‐T, the rate constant increased from 0.003 min^−1^ to 0.020 min^−1^, representing a 6.4‐fold increase. The Cr(VI) reduction performance of CN‐T was approximately 5.2 times that of CN. A concise comparison with recent studies focusing on the concurrent removal of phenol and Cr(VI) from coexisting systems is summarized in Table S2. Notably, compared with previously reported photocatalysts under similar reaction conditions, the CN‐T system does not require any additional sacrificial agents and can achieve a degradation rate of over 99% for phenol within 60 min. At the same time, phenol serves as an electron donor, significantly promoting the separation of photogenerated charges, and sustaining the reduction efficiency of hexavalent chromium at a relatively low level. This comparison highlights the distinct superiority of utilizing the pollutant itself as an intrinsic hole scavenger.

The valence band (VB) (Figure 1e) showed that CN‐T had a lower VB position than CN, which was consistent with its higher phenol degradation efficiency and improved the oxidizing capability of photogenerated h^+^ [30]. These results confirm CN‐T's superior photocatalytic oxidation‐reduction activity relative to CN, with phenol synergistically promoting Cr(VI) reduction without significant inhibition of its own degradation (>99.0%). 3D fluorescence spectroscopy (Figures S3 and 1f) confirmed stepwise phenol degradation via gradual quenching of characteristic aromatic peaks. Total organic carbon (TOC) analysis revealed that approximately 50% of phenol was mineralized after 60 min in the single system, while the coexistence of Cr(VI) led to a moderate reduction in mineralization efficiency to approximately 30% (Figure S4), consistent with the consumption of oxidative species in the simultaneous reduction process.

### 3.2 Mechanism of Electron Donor‐Mediated Synergism

**FIGURE 2:**
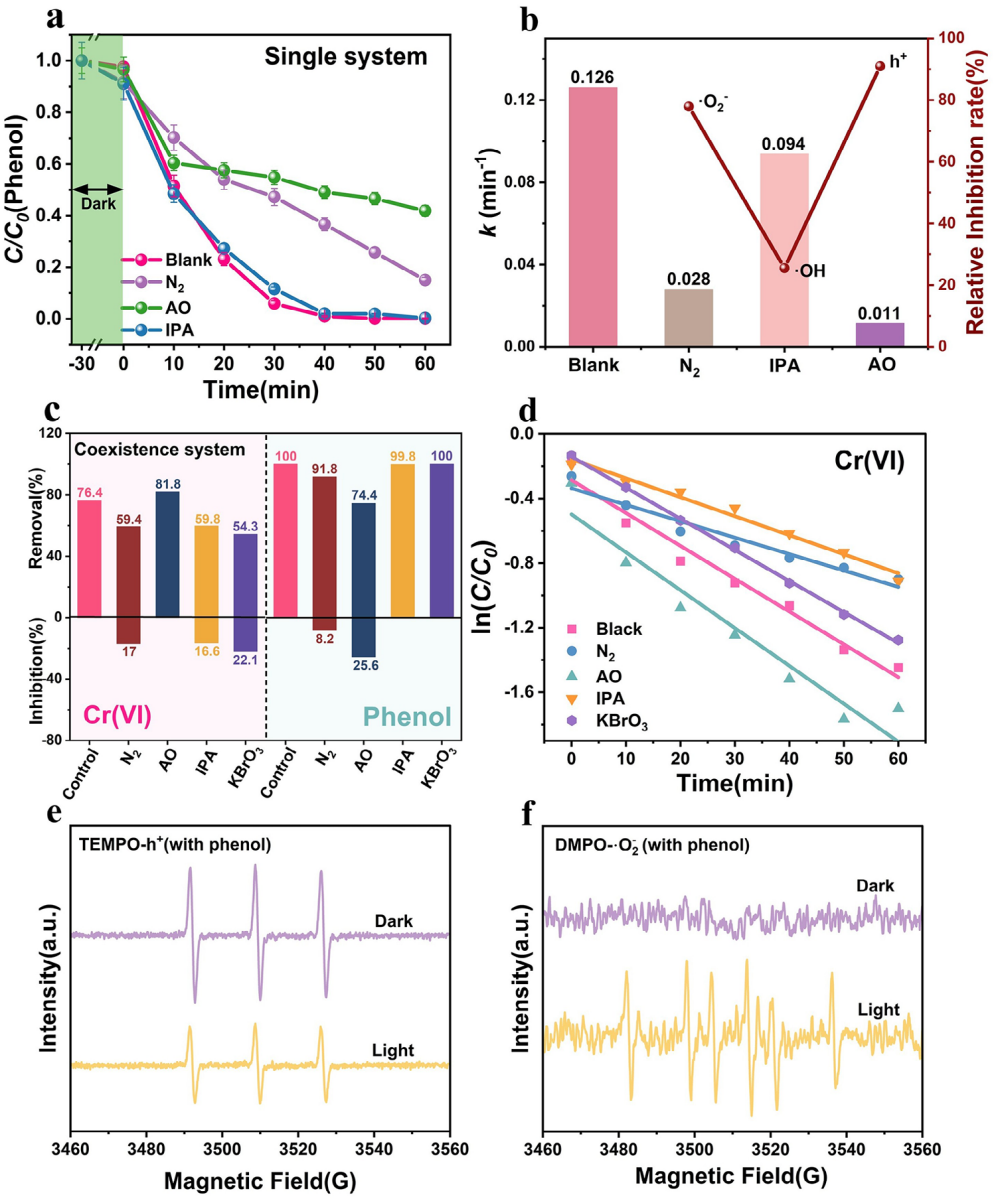
(a) Quenching effects on phenol degradation in single systems and (b) Corresponding active species contributions. The specific dosages of quenching agents are as follows: ammonium oxalate (AO, 5 mm), isopropanol (IPA, 5 mm), and potassium bromate (KBrO_3_, 5 mm). (c) Co‐removal efficiency under different quenchers and (d) Cr(VI) reduction rate constants. EPR spectra of CN‐T for (e) TEMPO‐h^+^ and (f) DMPO‐•O_2_^−^.

To clarify the synergistic mechanism between phenol and Cr(VI), radical quenching tests were carried out to systematically analyze the contributions of various reactive species. As scavengers for holes (h^+^), hydroxyl radicals (•OH), electrons (e^−^), and superoxide radicals (•O_2_^−^), ammonium oxalate (AO), isopropanol (IPA), KBrO_3_, and N_2_ were utilized, respectively. AO and N_2_ considerably inhibited degradation in the single phenol system, suggesting that h^+^ and •O_2_^−^ are the dominant reactive species (Figure 2a). On the other hand, IPA had a negligible inhibitory impact, indicating that •OH radicals are not very important in this system. Kinetic constant (Figure 2b) analysis showed that quenching h^+^ reduced the rate constant from 0.126 min^−1^ to 0.011 min^−1^ (inhibition rate 90.9%), while quenching •O_2_^−^ decreased it to 0.028 min^−1^ (inhibition rate 77.9%). The sum of their contributions exceeded 100%, suggesting a synergistic oxidation effect between h^+^ and •O_2_^−^. Specifically, h^+^ first converts phenol into phenoxy radicals and quinone intermediates, and •O_2_^−^ further attacks these intermediates to promote aromatic ring‐opening and mineralization. Thus, h^+^ and •O_2_^−^ act sequentially and synergistically, which is consistent with the well‐documented phenol degradation pathway in photocatalytic systems [24]. This synergistic feature is consistent with the common characteristics of multi‐species synergistic reactions in photocatalysis [31].

For Cr(VI) reduction, previous studies have suggested a reliance on direct photogenerated electron transfer. Herein, this pathway was validated by introducing KBrO_3_. In the coexistence system, KBrO_3_ reduced the Cr(VI) reduction rate by 22.1% but did not completely inhibit it, confirming that Cr(VI) reduction proceeds primarily via direct e^−^ capture. Notably, KBrO_3_ enhanced phenol degradation by 1.68‐fold (k = 0.127 min^−1^) (Figure S5), suggesting that e^−^ scavenging improves carrier separation and promotes h^+^‐mediated oxidation. Additionally, IPA decreased Cr(VI) reduction by 16.6%, implying that •OH competes with Cr(VI) for •O_2_^−^, further highlighting •O_2_^−^ as a critical bridge linking oxidation and reduction processes. EPR spectroscopy (Figure 2e,f) supported this mechanism: under light irradiation, the DMPO‐•O_2_^−^ signal intensified markedly relative to dark conditions, whereas the TEMPO‐h^+^ signal decreased, confirming •O_2_^−^ and h^+^ as the dominant reactive species. According to these results, phenol is analogous to methanol as a sacrificial agent for holes, thereby increasing the participation of photogenerated electrons from CN‐T in Cr(VI) reduction [32, 33].

**FIGURE 3:**
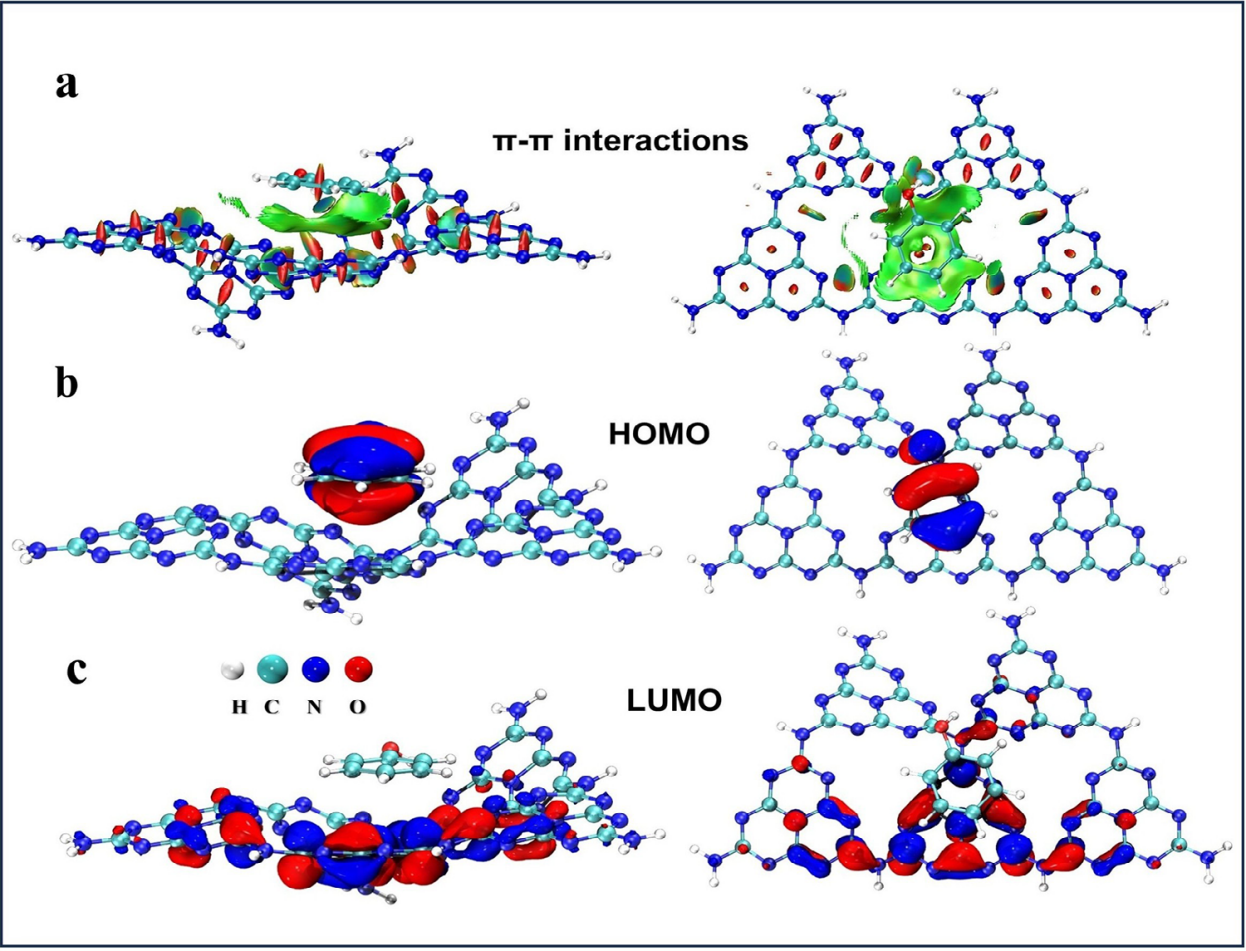
(a) π–π interactions and hydrogen bonding between phenol and CN‐T. (b) HOMO and (c) LUMO distributions in the phenol/CN‐T system.

To further elucidate phenol's electron‐donating mechanism, interfacial interactions and electron transport between phenol and CN‐T were examined using DFT simulations [34, 35]. Geometry optimization (Figure S6) yielded a stable binding motif in which the aromatic ring of phenol lies nearly parallel to the CN‐T basal plane at a van der Waals distance of 3.30 Å, while a N‐H···O contact (2.07 Å) anchors the hydroxyl group to a framework nitrogen site. Visualization of the non‐covalent interaction regions (Figure 3a) confirms that both π‐stacking and hydrogen‐bonding forces collectively ensure the stability of the adsorbate. According to frontier molecular orbital estimates (Figure 3b,c), the HOMO of phenol is higher in energy than that of CN‐T, whereas the LUMO of CN‐T is lower than that of phenol. This spatial isolation acts in concert with the HOMO–LUMO energy gap of the molecules, prompting the phenolic substances to inject electrons into the catalyst [36, 37, 38]. This computational model directly confirms the experimental inference, that is, the pollutant itself acts as a sacrificial agent for hole quenching, thereby releasing conduction band electrons to reduce hexavalent chromium to a lower valence state. Based on these findings, the synergistic mechanism in the coexistence system is proposed as follows:

(1) Phenol adsorbs onto CN‐T via π–π stacking and hydrogen bonding. Driven by the interfacial energy level difference, electrons transfer from phenol's HOMO to CN‐T's LUMO, consuming h^+^ and enriching photogenerated e^−^.

(2) Cr(VI) acts as a strong electron acceptor, capturing e^−^ from CN‐T to undergo reduction, accelerating e^−^/h^+^ separation and sustaining h^+^‐mediated phenol oxidation. This explains why phenol degradation remained >90.0% even under N_2_‐purged conditions (which suppress •O_2_^−^ generation).

**FIGURE 4:**
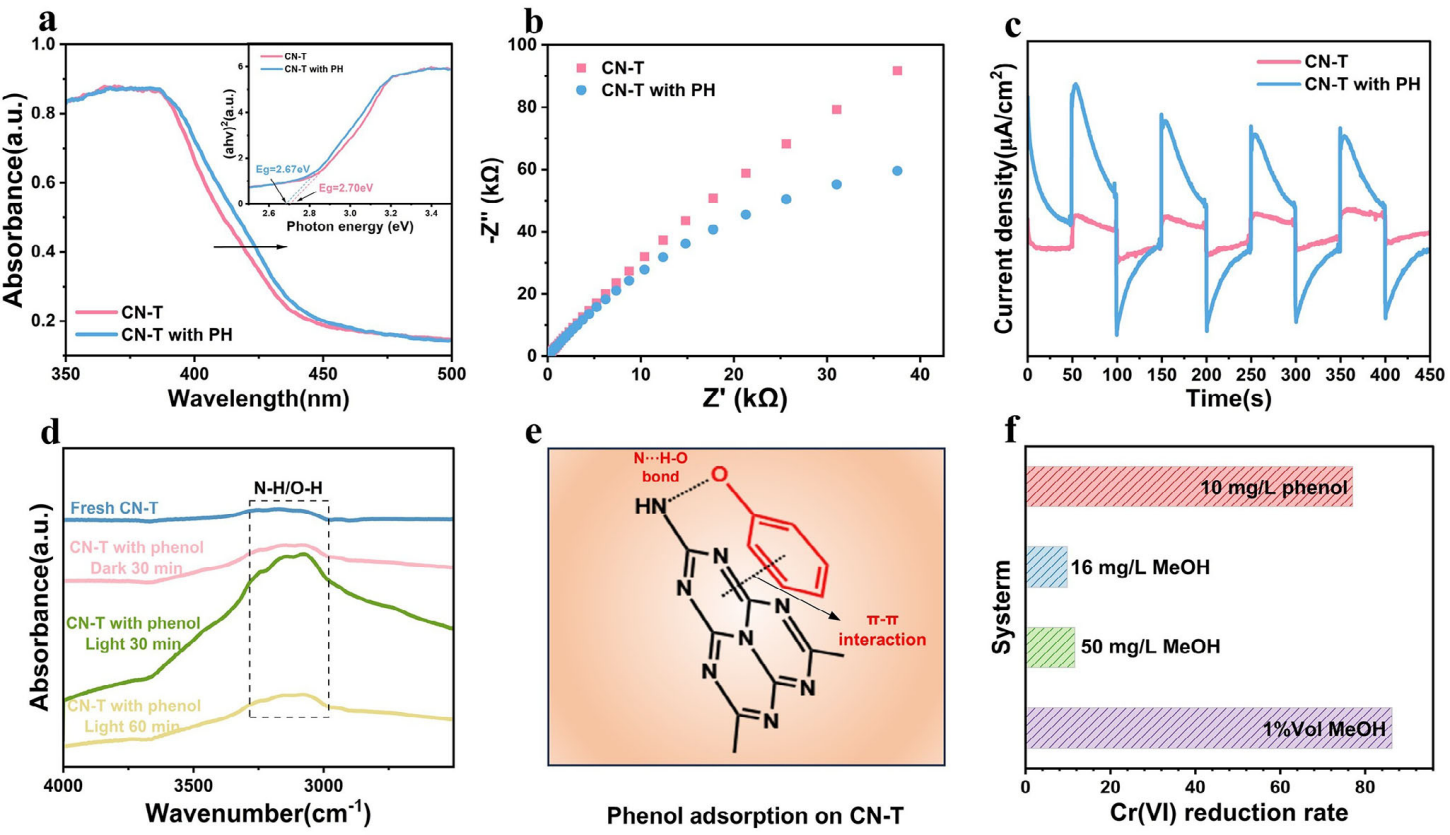
(a) UV–vis DRS spectra of CN‐T with and without phenol adsorption (inset: corresponding Tauc plots). (b) Nyquist plots and (c) photocurrent response of CN‐T with/without phenol. (d) FTIR spectra of CN‐T during phenol adsorption/degradation. (e) Schematic of phenol adsorption on CN‐T. (f) Comparison of the reduction rates of Cr (VI) by CN‐T under different electron donor systems.

Photoelectrochemical measurements further verified the enhanced charge separation induced by phenol adsorption. UV–vis DRS (Figure 4a) showed a slight bandgap narrowing from 2.70 eV to 2.67 eV after phenol adsorption, consistent with the electronic structure modulation via π–π stacking [39]. EIS Nyquist plots (Figure 4b) and Transient photocurrent (Figure 4c) confirmed that phenol adsorption accelerates charge separation and reduces interfacial resistance. The fitted charge transfer resistance (Rct) decreased sharply, and the photocurrent density increased notably, directly demonstrating the promoted charge separation and transfer efficiency [39, 40]. Dark‐state PL spectra of CN‐T before and after phenol adsorption are shown in Figure S7. The PL intensity of CN‐T after phenol adsorption is significantly quenched compared to pristine CN‐T, which directly confirms that phenol adsorption suppresses charge recombination by acting as an electron acceptor, providing strong evidence for the proposed charge separation mechanism. Finally, infrared spectroscopy tracked dynamic surface chemistry changes (Figure 4d,e) [41, 42], revealing the microscopic adsorption‐degradation mechanism: Pristine CN‐T displayed characteristic N─H (3066 cm^−1^) and O─H (3280 cm^−1^) stretching bands. Upon phenol adsorption under dark conditions, the O─H peak redshifted and broadened, signifying N─H···O hydrogen bond formation, while the triazine ring vibration shifted from 804 cm^−1^ to 808 cm^−1^, confirming π‐π stacking (Figure 4d) [43, 44]. After 30 min of irradiation, enhanced bands in the 1200–1625 cm^−1^ region indicated the accumulation of quinone and carboxylate intermediates (Figure S8). By 60 min, intermediate peaks disappeared, and the 3000–3300 cm^−1^ region returned to baseline, indicating effective destruction of the aromatic and functional groups in phenol and its intermediates, with gradual conversion toward CO_2_ and H_2_O.

These dynamic changes reveal the mechanism by which phenol acts. Phenol and its intermediate products, acting as electron donors, may further affect the charge distribution on the material surface through electron transfer, thereby enhancing the vibration signal of the N─H/O─H group. With the progress of the photocatalytic reaction, intermediate products are further degraded into small molecules such as CO_2_ and H_2_O. Interaction of the N─H/O─H groups weakens or is consumed, and the peak intensity decreases. Meanwhile, phenol's function as an electron donor diminishes progressively, and the electron transfer process on the material surface reaches a steady state, resulting in the vibration signal returning to a lower intensity. These dynamic changes not only reflect the intermediate steps of phenol degradation but also highlight phenol's critical function as an electron donor in the photocatalytic reaction. This dynamic evolution aligns with DFT‐calculated electron transfer pathways and EPR‐detected •O_2_^−^/h^+^‐dominated redox processes, confirming phenol's role as an electron donor in driving synergistic reactions.

Finally, to compare the performance of phenol with that of traditional sacrificial agents, a control experiment was conducted using methanol as a typical electron donor. We employed 16 mg/L methanol with the same electron molar concentration as 10 mg/L phenol, 50 mg/L methanol with a higher electron concentration, and 1% excess methanol by volume fraction. As shown in Figure 4f, the Cr(VI) reduction rate in the phenol system reached 77%, far higher than the 10% and 12% observed for 16 mg/L and 50 mg/L methanol, respectively. This indicates phenol serves as a far more effective electron donor than methanol at equal or higher electron equivalents. Although the excess methanol system presented a slightly higher removal rate, it relied on a large dosage of toxic sacrificial agent. In contrast, the CN‐T/phenol system needed no extra additives and employed phenol itself as the electron donor to achieve Cr(VI) reduction close to that of excess methanol, demonstrating high environmental compatibility and practical potential for real water remediation while avoiding secondary pollution and extra costs.

### 3.3 Effect of Environmental Factors

**FIGURE 5:**
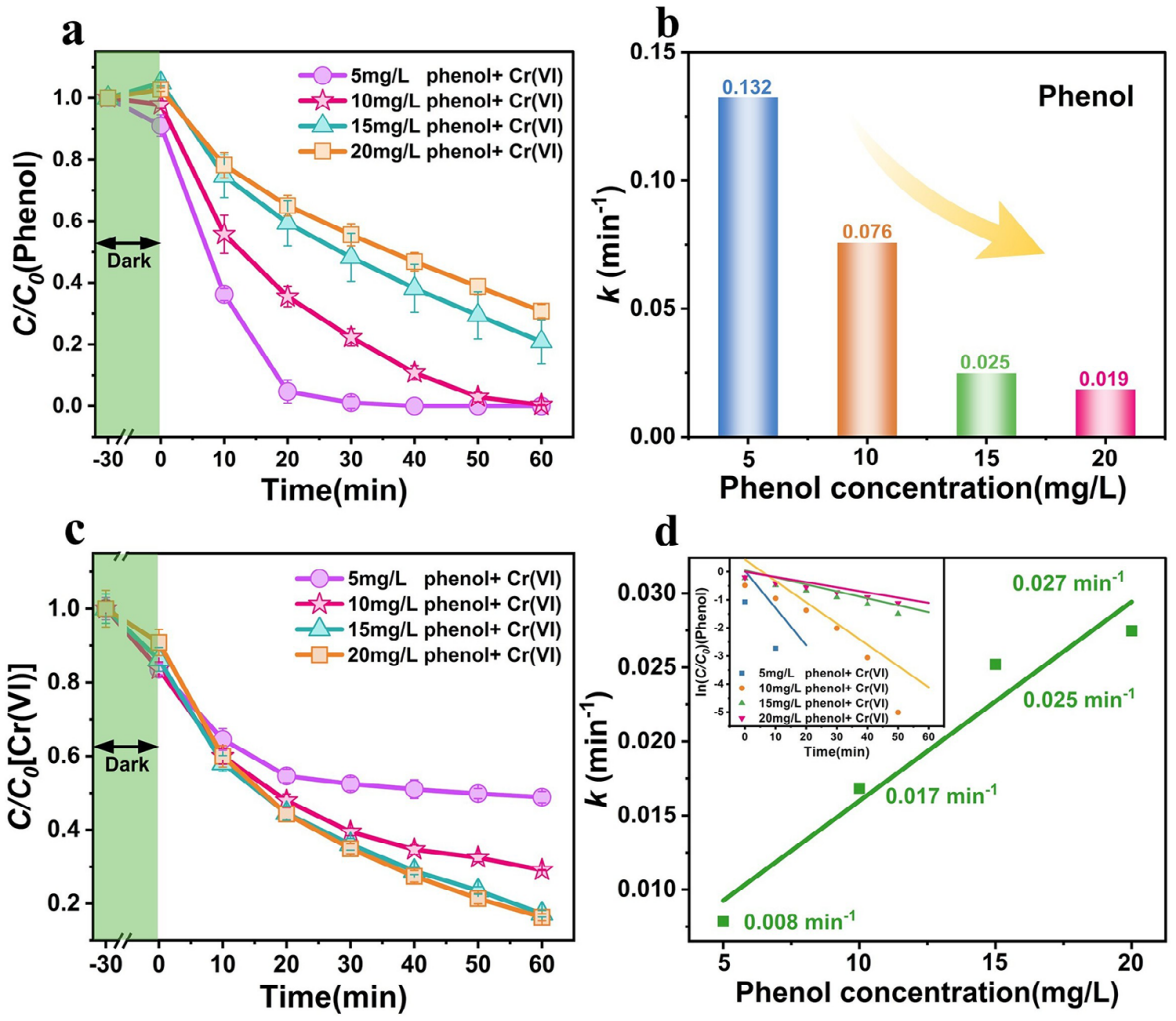
Influence of phenol concentration on (a) phenol degradation and (c) Cr (VI) reduction within the coexistence system. (b) Associated first‐order kinetic constants of phenol. (d) Linear correlation between the rate constant of Cr(VI) reduction and phenol concentration.

To assess how initial phenol concentration impacts Cr(VI) reduction, experiments were performed with phenol concentrations ranging from 5 mg/L to 20 mg/L, while the Cr(VI) concentration was kept fixed at 5 mg/L. Phenol degradation efficiency dropped from approximately 100% to 67.2% over the course of 60 min when varying the initial phenol concentration from 5 mg/L to 20 mg/L, as illustrated in Figure 5a,b. Correspondingly, the pseudo‐first‐order rate constant (k) exhibited a downward trend, falling from 0.132 min^−1^ to 0.019 min^−1^ with increasing phenol. Notably, 99.8% degradation was maintained at 10 mg/L phenol (k = 0.126 min^−1^), suggesting substrate saturation limits electron transfer at higher concentrations. When the initial phenol concentration reached 20 mg/L, the removal rate of phenol was only 67.2% after 60 min. This might be because phenol and Cr(VI) are not only the target pollutants but also necessary factors for inducing the reaction [45]. However, in this reaction system, with a fixed initial Cr(VI) concentration, electron transfer is limited at elevated phenol concentrations. Conversely, as phenol content increased, Cr(VI) reduction effectiveness considerably increased (Figure 5c,d). At a phenol concentration of 20 mg/L, the reduction of Cr(VI) reached 83.7% (k = 0.027 min^−1^), which is a 3.5‐fold increase compared to a 5 mg/L concentration of phenol. The strong positive correlation (R^2^ = 0.92) between k and phenol concentration confirms phenol's role as an electron donor, where higher concentrations promote e^−^/h^+^ separation and accelerate Cr(VI) reduction.

**FIGURE 6:**
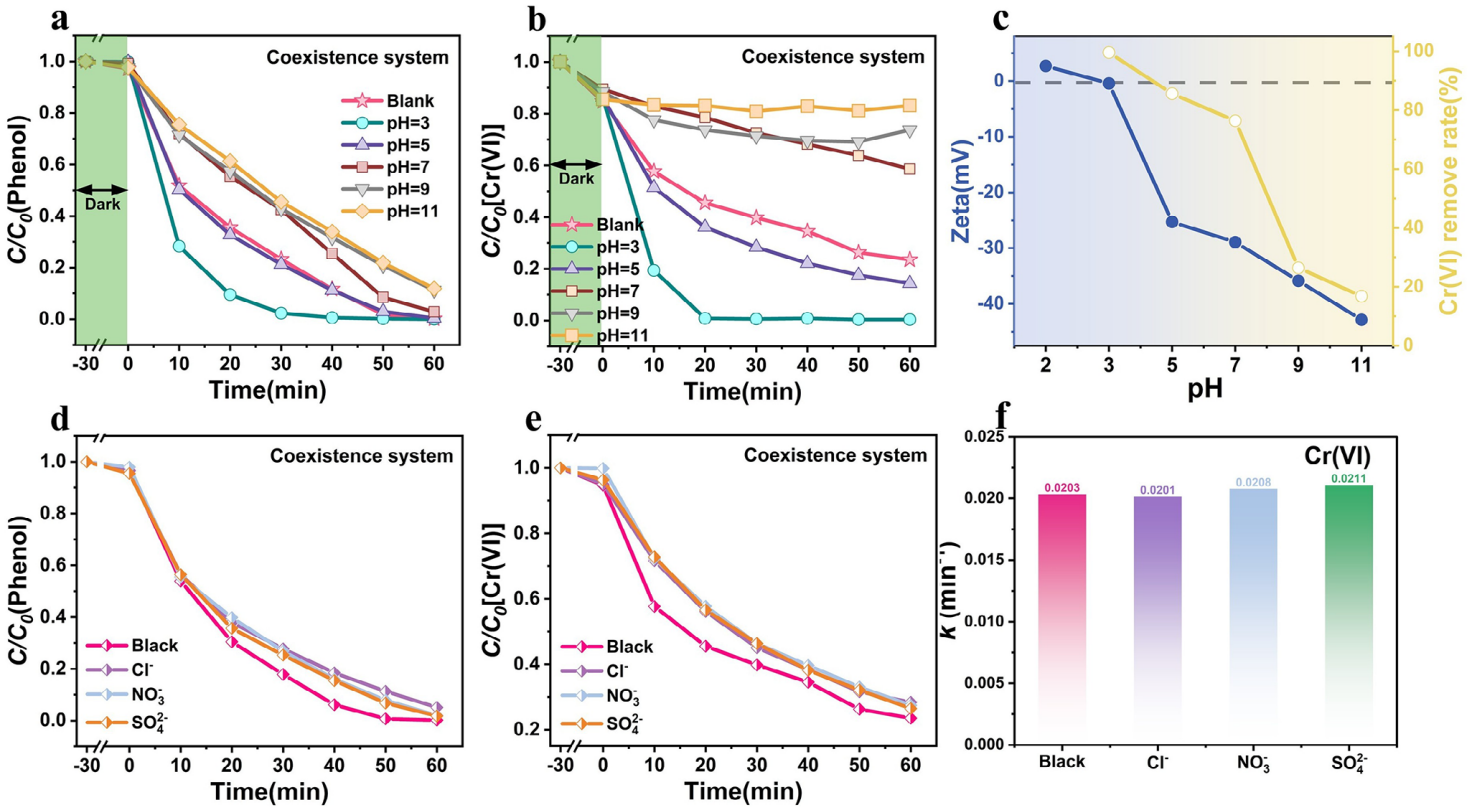
The influence of pH value on the removal of (a) phenol and (b) Cr(VI) in a coexisting system. (c) Zeta potential of CN‐T at different pH levels in coexistence systems. Effects of common inorganic anions on (d) phenol degradation and (e) Cr(VI) reduction in the coexisting system. (f) Corresponding pseudo‐first‐order rate constants of Cr(VI) reduction in the presence of different anions.

Solution pH was found to critically influence reaction kinetics, as it modulates pollutant speciation and catalyst surface charge [32, 46]. In this study, the influence of pH on the removal behavior of phenol and Cr(VI) was systematically investigated. As shown in Figure S9 and Figure 6a, in both single and coexisting systems, phenol degradation efficiency decreased significantly with increasing pH. A weakly acidic environment (pH 3.0) was most conducive to phenol degradation, with the degradation rate constant rising from 0.092 min^−1^ at pH 7.0 to 0.266 min^−1^ at pH 3.0. However, in alkaline solutions, the phenol removal effect decreases, indicating that a weakly acidic environment is more conducive to oxidative degradation of phenol. Both protonation and ionization states of pollutants affect the photocatalytic reaction rate constant. Since the pKa value of phenol is 9.89 (Figure S9c), under acidic conditions, phenol exists as a neutral molecule, and the hydrogen on its phenolic hydroxyl group is not easily ionized. Under alkaline conditions, solution pH is higher than the pKa value of phenol, causing phenol to ionize and form oxygen anions [47]. Furthermore, in strongly acidic solutions, a high H^+^ concentration results in protonation of the CN‐T surface. The protonated CN‐T surface carries a positive charge, with protonated amino groups (‐NH_3_^+^) serving as hydrogen bond donors and forming N‐H···O hydrogen bonds with hydroxyl oxygen atoms of phenol, accelerating transfer of photogenerated electrons to the CN‐T surface. Meanwhile, the electron‐rich π system of CN‐T and the aromatic ring of phenol produce a π–π stacking effect, synergically promoting the formation of electron delocalization and interfacial transport channels, jointly optimizing the electron transfer path and enabling phenol to be oxidized by holes more rapidly [48]. In contrast, alkaline conditions lead to phenol ionization into phenoxide anions, reducing benzene ring electron cloud density, and the negatively charged CN‐T surface (Zeta potential < 0 when pH > 3.0) generates electrostatic repulsion with phenoxide anions, inhibiting adsorption and electron transfer.

Figure 6b shows that acidic conditions (pH 3.0, 5.0) are more conducive to synergistic Cr(VI) reduction than alkaline conditions (pH 9.0, 11.0). As shown in Figure S9d, speciation diagrams indicate that, below pH 7.0, Cr(VI) primarily exists as ${\mathrm{HCrO}}_{4}^{-}$ or $Cr_{2}O_{7}^{2-}$, whereas at neutral and alkaline pH, it chiefly exists as ${\mathrm{CrO}}_{4}^{2-}$. ${\mathrm{Cr}}_{2}O_{7}^{2-}\left[E\left({\mathrm{Cr}}_{2}O_{7}^{2-}/{\mathrm{Cr}}^{3+}\right)=+1.36\mathrm{eV}\;\mathrm{vs}.\mathrm{NHE}\right]$ is more easily reduced to Cr^3+^ than ${\mathrm{CrO}}_{4}^{2-}\left[E\left({\mathrm{CrO}}_{4}^{2-}/\mathrm{Cr}{\left(\mathrm{OH}\right)}_{3}\right)=-0.13\;\mathrm{eVvs}.\mathrm{NHE}\right]$. Under acidic conditions, excess H^+^ promotes the reduction of ${\mathrm{HCrO}}_{4}^{-}$ or ${\mathrm{Cr}}_{2}O_{7}^{2-}$ through the following reaction Equations (4) and (5).

Under acidic conditions:

(4) $${\mathrm{HCrO}}_{4}^{-}+7H^{+}+3e^{-}\to Cr^{3+}+4H_{2}O$$

(5) $$Cr_{2}O_{7}^{2-}+14H^{+}+6e^{-}\to 2Cr^{3+}+7H_{2}O$$

However, in neutral and alkaline systems, high concentrations of hydroxide ions (OH^−^) hinder the reduction process of Cr $O_{4}^{2-}$ ions (Equation 6).

Under neutral and alkaline conditions:

(6) $${\mathrm{CrO}}_{4}^{2-}+4H_{2}O+3e^{-}\to \mathrm{Cr}{\left(\mathrm{OH}\right)}_{3}+5OH^{-}$$

Consistent with this speciation‐dependent reactivity, the present study observed maximal Cr(VI) removal at pH 3.0, with efficiency declining sharply as pH increased beyond the point of zero charge of CN‐T.

For example, at pH 5.0, Cr(VI) reduction efficiency is only 31.4%. In the coexistence system, even under natural pH 6.3, Cr(VI) can still achieve a reduction efficiency of 76.4%. This demonstrates that phenol's presence facilitates Cr(VI) reduction over a wide pH range. As pH rises to the alkaline range, the dominant species of Cr(VI) in aqueous media becomes Cr $O_{4}^{2-}$, which is difficult to reduce even in the coexisting system with phenol. The regulatory mechanism of CN‐T surface charge state on Cr(VI) reduction was further revealed through Zeta potential analysis (Figure 6c). The zero charge point of CN‐T is 3.0. When the solution pH is greater than 3.0, the CN‐T surface carries a negative charge; when the pH is less than 3.0, the surface carries a positive charge, and the Zeta potential of CN‐T gradually decreases with increasing pH. Therefore, in acidic solutions, CN‐T provides a favorable environment for the adsorption of ${\mathrm{HCrO}}_{4}^{-}$ ions. In alkaline solutions, Cr(VI) mainly exists as ${\mathrm{CrO}}_{4}^{2-}$ and $Cr_{2}O_{7}^{2-}$. Due to electrostatic repulsion, CN‐T with a relatively large negative charge is not conducive to the adsorption of ${\mathrm{CrO}}_{4}^{2-}$ and $Cr_{2}O_{7}^{2-}$.

Beyond solution pH, inorganic anions ubiquitously present in natural waters and industrial effluents represent another critical factor affecting the practical applicability of photocatalytic systems, as they may scavenge reactive species or compete for active sites on the catalyst surface. Herein, the effects of the three most prevalent anions in actual water bodies (Cl^−^, SO_4_^2−^, NO_3_^−^) on the simultaneous phenol degradation and Cr(VI) reduction over CN‐T were systematically evaluated. The anion concentrations were set in accordance with the typical background levels commonly adopted in photocatalytic studies: 18.7 mg/L Cl^−^, 0.2 mM SO_4_^2−^, and 0.1 mM NO_3_^−^.

As shown in Figure 6d–f, Cl^−^ exhibits a moderate inhibitory effect on the degradation of phenol. However, after 60 min of irradiation, the removal efficiency still remained above 94%. This moderate inhibitory effect of chloride ions can be attributed to their scavenging of photogenerated h^+^, which act as the primary reactive species involved in the oxidation of phenol in this system. In contrast, the presence of these anions has almost no effect on the reduction of Cr(VI). In the presence of Cl^−^, NO_3_^−^, and SO_4_^2−^, the pseudo‐first‐order kinetic constants for Cr(VI) reduction are 0.020 min^−1^, 0.021 min^−1^, and 0.021 min^−1^, respectively, with no notable difference compared to the blank control (0.020 min^−1^). Overall, these findings demonstrate that the proposed system maintains high stability under various water chemical conditions. It is noteworthy that its tolerance to coexisting ions and its relatively broad pH range adaptability highlight its potential applicability in complex wastewater systems.

### 3.4 Practical Applicability to Industrial Wastewater

It is of great significance to verify the performance of the photocatalytic system under realistic industrial pollutant concentrations. In industrial wastewater, phenol is widely distributed in coking, petrochemical, printing and dyeing, and pharmaceutical wastewater. The raw water concentration ranges from 50 to 500 mg/L, and usually reduces to 10–50 mg/L after primary biological pretreatment 57. Cr(VI) commonly exists in electroplating, leather tanning, metallurgy, and chemical wastewater, with a raw water concentration of 20–200 mg/L and 5–50 mg/L after pretreatment 58. The phenol (10 mg/L) and Cr(VI) (5 mg/L) concentrations adopted in this study are within the above typical ranges, which can well represent the actual working conditions.

The self‐sustaining mechanism proposed in this work depends on phenol as an intrinsic electron donor to promote Cr(VI) reduction. When phenol is sufficient, this mechanism can operate efficiently without any exogenous sacrificial agents. At relatively low pollutant concentrations, the electron‐donor‐mediated redox pathway still holds true, but the overall reaction rate may decrease due to the limited electron donor supply. In actual complex wastewater, various coexisting organic matters can jointly act as electron donors to maintain the redox cycle. Therefore, the intrinsic electron donor‐driven synergistic mechanism proposed in this work is not limited to ideal laboratory conditions and has good potential for application in real industrial wastewater, although the efficiency will be affected by pollutant composition and concentration.

### 3.5 Substituent Effects and Structure‐Activity Relationships

**FIGURE 7:**
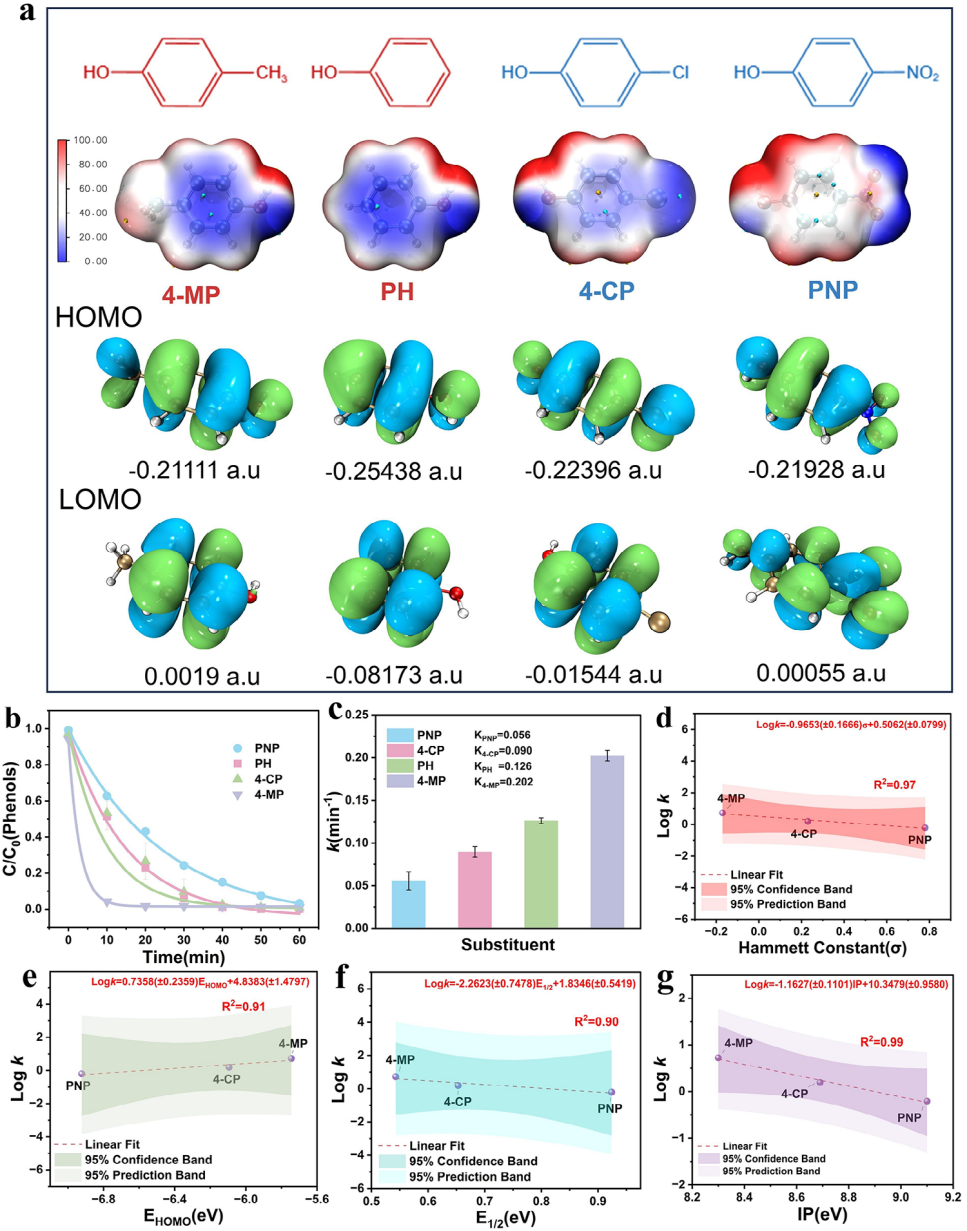
(a) Electrostatic potential (ESP) distribution and HOMO LUMO orbitals of 4‐MP, PH, 4‐CP, and PNP. (b) Degradation curves and (c) rate constants for substituted phenols. Correlations of Log k to (d) Hammett Constant (σ), (e) E_HOMO_, (f) E_1/2,_ and (g) IP.

Traditional photocatalytic processes based on radical oxidation often depend on excessive catalysts or oxidants to attain effective pollutant removal. In contrast, this study proposes to utilize pollutants themselves as electron donors to promote synergistic degradation of coexisting pollutants, thereby reducing unnecessary oxidant consumption and achieving “waste treatment with waste”. To clarify the superiority of this system, phenolic compounds with different para‐substituents were selected, including p‐chlorophenol (4‐CP), p‐nitrophenol (PNP), and p‐cresol (4‐MP), and the removal effect of CN‐T on these phenolic compounds was explored. As shown in Figure 7a, these monocyclic aromatic compounds have the same benzene ring structure, so their chemical properties and activities mainly depend on substituent electronic effects. All three para‐substituted phenols contain the same hydroxyl group (‐OH). At the same concentration (10 mg/L) and after 60 min of photocatalytic degradation, all four aromatic compounds reached 99% degradation. The degradation kinetics of the para‐substituted phenols showed a clear correlation with the electronic nature of the substituent, with the derived pseudo‐first‐order rate constants following the sequence: 4‐MP (k = 0.202 min^−1^) > phenol (k = 0.126 min^−1^) > 4‐CP (k = 0.090 min^−1^) > PNP (k = 0.056 min^−1^) (Figure 7b,c). This reactivity trend aligns with the electron‐donating/withdrawing capacity of the substituent group. Specifically, the electron‐donating methyl group enriches the π‐electron density of the aromatic ring, thereby enhancing its susceptibility to oxidative attack by photogenerated holes and facilitating the initial electron transfer step. Conversely, the strong electron‐withdrawing nitro group depletes ring electron density, rendering p‐nitrophenol significantly less reactive toward hole‐mediated oxidation. This trend underscores the pivotal role of the pollutant's intrinsic electronic structure in dictating its efficiency as an in situ hole scavenger within the dual‐substrate photocatalytic system.

To rationalize the substituent‐dependent reactivity trends observed experimentally, a quantitative structure–activity relationship (QSAR) analysis was undertaken. The electronic influence of each para‐substituent was quantified using four well‐established molecular descriptors: (i) the Hammett constant (σ), which is a parameterized description of the induction effect and resonance effect obtained through experimental methods [49]; (ii) the vertical ionization potential (IP), a measure of the energy required for electron abstraction [50]; (iii) the energy of the highest occupied molecular orbital (E_HOMO_), which is associated with susceptibility to electrophilic attack; and (iv) the half‐wave oxidation potential (E_1/2_) [51]. Collectively, these descriptors provide a multi‐faceted assessment of the electron‐donating or withdrawing properties of the substituent group and its resulting influence on hole‐mediated oxidation [52, 53]. Detailed data on σ, IP, E_HOMO_, and E_1/2_ are provided in Figure 7a and Table S3.

Organic pollutants with EDG (such as 4‐MP and PH) exhibit lower σ values and are more conducive to sacrificing electrons for oxidation. Conversely, organic pollutants with EWG (such as 4‐CP, PNP) have higher σ values and are not conducive to electron transfer for oxidation [52]. Theoretical descriptors such as IP, E_HOMO,_ and E_1/2_ can also correlate with the oxidation potential of organic pollutants. These theoretical descriptors are obtained through theoretical calculations and are applicable to any compound with a known chemical structure. As a result, they usually probe the mechanistic basis of the origin of selective oxidation [54]. Combined with the electrostatic potential analysis of phenols, EDG (such as ‐CH_3_) expands the negative potential distribution of the benzene ring. Conversely, EWGs such as ‐NO_2_ and‐Cl reduce electron density of the benzene ring, weakening its electron‐donating capacity and thereby decreasing its degradation activity. Accordingly, phenols substituted with EDG (e.g., 4‐MP) are expected to exhibit higher degradation activity than those with EWG, with the predicted activity trend: ‐CH_3_> ‐Cl > ‐NO_2_.

To verify the above speculation, the degradation rate constant k_PH_ of phenol was taken as the reference. Relative rate constants of other phenols were calculated using Equation (7). A quantitative structure‐activity relationship was established between Log k and structural parameters (σ, IP, E_HOMO_ or E_1/2_) to further explore the reaction mechanism and predict reaction rates of other organic compounds [55, 56]. A quantitative correlation between the observed degradation rate constants and the computed molecular descriptors was established (Figure 7d–g; Table S3). Among the parameters evaluated, the ionization potential (IP) exhibited the strongest linear correlation with log k (R^2^ = 0.99), outperforming the Hammett constant (σ, R^2^ = 0.97), E_HOMO_ (R^2^ = 0.91), and E_1/2_ (R^2^ = 0.90). These findings strongly suggest that the electronic properties of substituents play a crucial role in governing the oxidation kinetics of phenolic compounds, and the electron donor effect of EDG is pivotal in accelerating the oxidation reaction rate.

(7) $$\mathrm{Log}\;k=\;\mathrm{Log}\frac{k_{phenols}}{k_{PH}}$$

**FIGURE 8:**
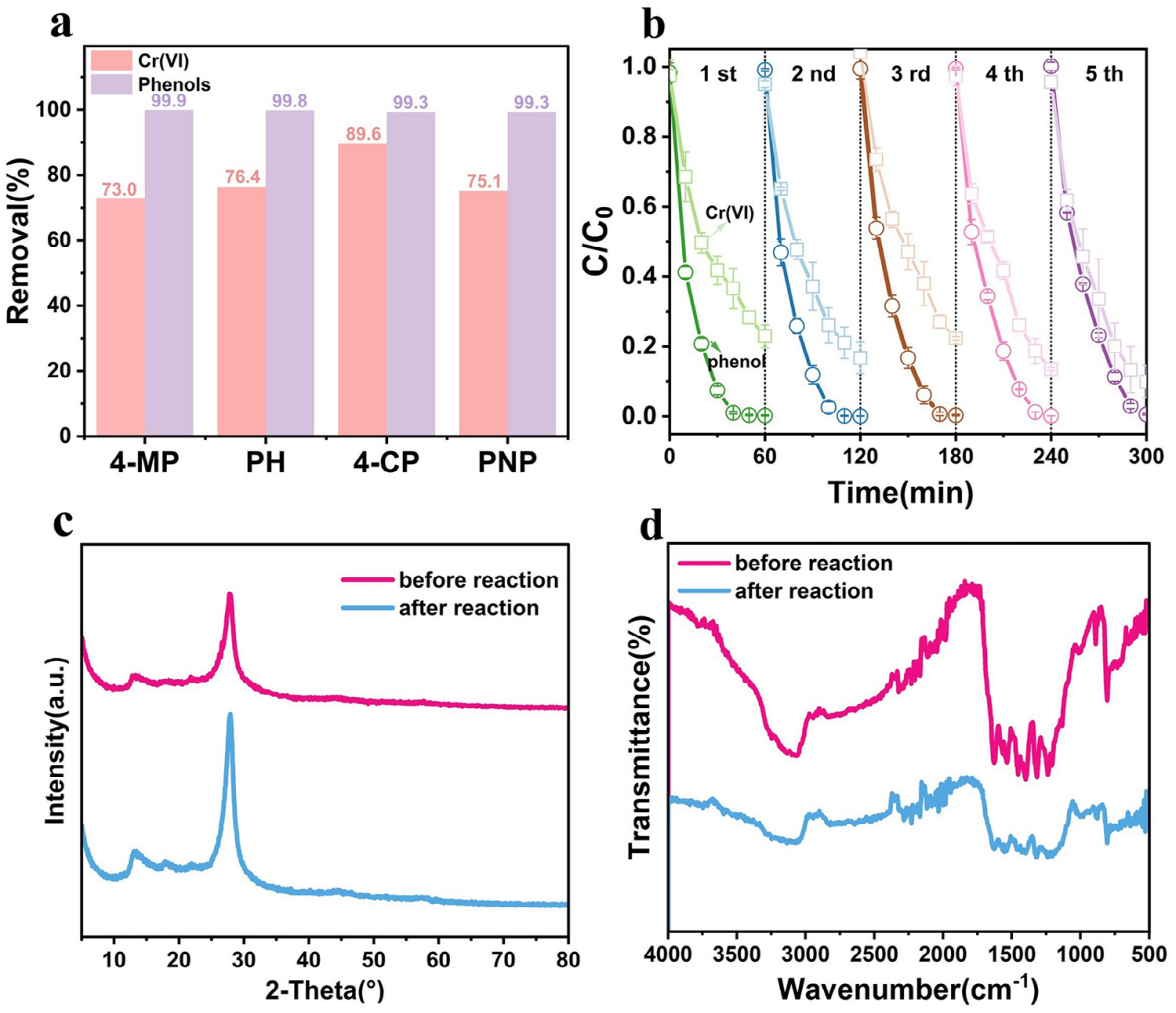
(a) Removal efficiencies of substituted phenols and Cr(VI) in coexistence systems. (b) Cycling performance of CN‐T for synchronous phenol and Cr(VI) removal. (c) XRD and (d) FT‐IR spectra of CN‐T before and after cycling.

Furthermore, this study explored pollutant removal effects in coexistence systems of different phenols and Cr(VI) (Figure 8a). Results show that under coexistence conditions, PH, 4‐CP, PNP, and 4‐MP all maintain high removal rates, while Cr(VI) reduction rates show certain differences. Among them, when 4‐MP coexists with Cr(VI), Cr(VI) reduction efficiency is the lowest. In the coexistence system, with PH and PNP, Cr(VI) reduction efficiency is relatively close. In summary, the order of effects of phenol and its para‐substituted phenols on reduction and removal of Cr(VI) in the coexisting system is: 4‐CP > PH > PNP > 4‐MP. Further analysis (Figure S11a) revealed that the degradation rate of 4‐MP reached 99.0% at 10 min. It is speculated that after 10 min of reaction, due to almost complete degradation of 4‐MP in the system, hole consumption decreased, thereby slowing down the Cr(VI) reduction rate. A similar phenomenon was also observed in the phenol system (Figure S11b), showing the same trend. When the concentration of phenol in the coexisting system was 5 mg/L, 30 min later, phenol was almost completely degraded, and the reduction rate of Cr(VI) significantly slowed down, showing the same trend. These substituent‐dependent reactivity trends further corroborate the proposed electron‐donor‐mediated mechanism, in which electron‐donating groups facilitate hole consumption and promote electron transfer for Cr(VI) reduction. From a practical perspective, utilizing pollutants as intrinsic hole sacrificial agents reduces the reliance on external oxidants, offering a more sustainable and potentially scalable strategy for complex wastewater treatment.

After five consecutive cycles of synchronous degradation of phenol and Cr(VI), the CN‐T catalyst exhibited excellent reusability and stability. The phenol degradation efficiency remained above 99% within 60 min in all cycles (Figure 8b). The reduction efficiency of Cr(VI) remained stable at around 80%, and slightly increased with the number of cycles (Figure S12b). This might be due to the phenol degradation intermediates adsorbed on the catalyst surface during the cycling process, serving as in situ electron donors, which continuously strengthened the interface charge transfer. XRD and FTIR characterizations confirmed that the crystal structure and surface functional groups of CN‐T were well preserved after cycling, indicating outstanding structural and catalytic stability. These results provide a solid basis for the practical application of this “waste to waste” remediation strategy (Figure 8c,d).

## 4 Conclusions

The coexistence of organic pollutants and heavy metals in industrial wastewater demands sustainable and efficient remediation technologies. In this work, we develop an exogenous oxidant/sacrificial agent‐free dual‐substrate system where phenol acts as an intrinsic electron donor to drive synergistic phenol degradation and Cr(VI) reduction over CN‐T. Key innovations include:

(1) **Novel Synergistic mechanism**: Phenol acts as an electron donor, adsorbing onto CN‐T via π–π stacking and hydrogen bonding, enabling directed electron transfer from phenol's HOMO to CN‐T's LUMO. This transfer consumes photogenerated h^+^, thus promoting phenol degradation and enriching e^−^ for enhanced Cr(VI) reduction, while suppressing e^−^‐h^+^ recombination, eliminating the need for external reagents and achieving “waste‐to‐waste” remediation.

(2) **Exceptional performance enhancement**: In the coexisting system, CN‐T achieves 99.8% phenol degradation and 76.4% Cr(VI) reduction. The Cr(VI) reduction rate constant is 6.4 times higher than that in the single Cr(VI) system, and CN‐T outperforms CN by around 5.2‐fold in Cr(VI) reduction—demonstrating the critical role of catalyst modification and dual‐substrate synergy.

(3) **Environmental factor and substituent effects**: Acidic conditions (pH 3.0) are optimal for both phenol degradation and Cr(VI) reduction, as they promote phenol adsorption and Cr(VI) speciation into easily reducible ${\mathrm{HCrO}}_{4}^{-}/Cr_{2}O_{7}^{2-}$. For substituted phenols (4‐MP, 4‐CP, PNP), reactivity correlates strongly with substituent electronic properties (Hammett σ, E_HOMO_, ionization potential [IP]), with electron‐donating groups (e.g., ‐CH_3_) enhancing degradation efficiency. Quantitative structure‐activity relationships (QSARs) with R^2^> 0.90 are established, providing a predictive framework for synergistic co‐removal of other organic‐heavy metal pairs.

(4) **Mechanistic insight via multi‐technique validation**: EPR spectroscopy confirms h^+^ and •O_2_^−^ as dominant active species for phenol oxidation, while DFT calculations and transient photocurrent measurements clarify electron transfer pathways and charge separation efficiency. FTIR and 3D fluorescence spectroscopy track phenol adsorption, intermediate formation, and mineralization—validating the dynamic evolution of the photocatalytic process.

This work advances fundamental knowledge of photocatalytic redox coupling and offers a sustainable, cost‐effective solution for complex wastewater treatment. The exogenous reagent‐free design and predictive QSARs provide a blueprint for scaling this technology to real‐world industrial pollution control, where organic and heavy metal pollutants coexist. Future efforts will focus on optimizing the catalyst structure for broader pollutant adaptability and evaluating the system's performance in large‐scale pilot tests, further promoting its practical translation.

## Conflicts of Interest

The authors declare no conflict of interest.

## Supporting information




**Supporting File**: advs75763‐sup‐0001‐SuppMat.docx.

## Data Availability

The data that support the findings of this study are available from the corresponding author upon reasonable request.
