# Supplementary material for: Dual‐Substrate Synergistic Photocatalysis: Exogenous Reagent‐Free Co‐Removal of Phenol and Cr(VI) via Electron‐Donor‐Mediated Redox Coupling over Modified Carbon Nitride
Source: Adv Sci (Weinh). 2026 Jun 1:e75763. Online ahead of print. doi: 10.1002/advs.75763 (PMC13335979; doi:10.1002/advs.75763)
Supplement: Supplementary file 1 — Supporting File: advs75763‐sup‐0001‐SuppMat.docx. [file ADVS-9999-e75763-s001.docx]

**Supporting Information**

**Dual-Substrate Synergistic Photocatalysis: Exogenous Reagent-Free Co-Removal of Phenol and Cr(VI) via Electron-Donor-Mediated Redox Coupling over Modified Carbon Nitride**

Xiaoman Zhang ^a^, Qiyu Wang ^a^, Xiaodan Liu ^a^, Shuqi Wan ^a^, Cuiwei Du ^a^, Qilu Li ^a^, Chongfei Yu ^a^, Shuying Dong ^a,^^^[[1]](#footnote-1)^^ , Xianfa Su ^a,^[[2]](#footnote-2)^^ , Chun Hu ^a,b^[[3]](#footnote-3)^^

*^a^ School of Environment, Henan Normal University, Key Laboratory for Yellow River and Huai River Water Environmental and Pollution Control, Ministry of Education, Henan Key Laboratory for Environmental Pollution Control, Xinxiang, Henan, 453007, P.R. China*

*^b^ Institute of Environmental Research at Greater Bay, Key Laboratory for Water Quality and Conservation of the Pearl River Delta, Ministry of Education, Guangzhou*

*University, Guangzhou 510006, PR China*

Summary

[Text S1. Chemicals and reagents 4](#_Toc227282433)

[Text S2. Catalyst synthesis and characterizations 4](#_Toc227282434)

[Text S3. Theoretical calculation. 7](#_Toc227282435)

[Table S1 Basic Information on Substituted Phenols. 9](#_Toc227282436)

[Table S2. Comparison of photocatalytic removal of Phenol and Cr(VI) with recently reported catalysts. 10](#_Toc227282437)

[Table S3. The relevant parameters for para-substitute organics degradation. 11](#_Toc227282438)

[Fig. S1. Removal of Phenol and Cr(Ⅵ) without CN-T catalysts in (a) single and (b) coexistence system. 12](#_Toc227282439)

[Fig. S2. Comparison diagram of catalyst dosage for 10 mg/L phenol degradation. 13](#_Toc227282440)

[Fig. S3. 3D EEMs of phenol in the coexistence system: (a) the original solution; (b-h) collected after irradiation times of 0, 10, 20, 30, 40, 50 and 60 min, respectively;(i) DI water. 14](#_Toc227282441)

[Fig. S4. TOC removal efficiencies of phenol in single and coexistence systems after 60 min photocatalysis. 15](#_Toc227282442)

[Fig. S5. Comparison of phenol degradation rates with and without KBrO_3_ in coexistence system. 16](#_Toc227282443)

[Fig. S6. The optimized structure of Phenol/CN-T system (white, H; gray, C; blue, N; red, O). 17](#_Toc227282444)

[Fig. S7. PL spectra of pristine CN-T and CN-T after phenol adsorption. 18](#_Toc227282445)

[Fig. S8. FTIR spectra of CN-T before and after adsorption of phenol and at different degradation time. (b) FTIR spectra after adsorption of varying phenol concentrations. 19](#_Toc227282446)

[Fig. S9. The influence of pH value on the removal of (a) phenol and (b) Cr(VI) in a single system. (c)Existence patterns of phenol in different pH solutions. (d) Cr(VI) speciation at different pH. 20](#_Toc227282447)

[Fig. S10. In the absence and presence of typical anions, a pseudo-first-order model was fitted for the (a) phenol degradation and (b) Cr(VI) reduction on CN-T. (c) Corresponding pseudo-first-order rate constants (k) of phenol degradation under different anion conditions. 21](#_Toc227282448)

[Fig. S11. (a) Degradation activity of 4-MP and Cr(VI) in a coexisting system for 10 min. (b) Degradation performance of 5 mg/L phenol and Cr(VI) in a coexisting system. 22](#_Toc227282449)

[Fig. S12. (a) Co-removal curves for substituted phenols and Cr(VI) in coexistence systems. (b) Final removal efficiencies of phenol and Cr(VI) over CN-T during 5 consecutive cyclic runs. 23](#_Toc227282450)

[Fig. S13. (a) ESR spectra. (b) N₂ adsorption-desorption isotherms. 24](#_Toc227282451)

[References 25](#_Toc227282452)

# Text S1. Chemicals and reagents

All reagents were of analytical grade (≥99.0% purity) and used without further purification. Dicyandiamide was obtained from Aladdin Reagent Co., (China). Ethanol (EtOH) was purchased from Tianjin Chemical Reagent Co., Ltd. (Tianjin, China). Isopropyl Alcohol (IPA) was purchased from Tianjin Deen Chemical Reagent Co., Phenol, 4-Chlorophenol (4-CP), p-Nitrophenol (PNP), 4-Methylphenol (4-MP), Furfuryl alcohol (FFA), Ammonium oxalate (AO) were bought from Shanghai Aladdin Biochemical Technology Co., Ltd. Potassium dichromate was purchased from Zhengzhou Paini Chemical Reagent Factory. 5,5-dimethyl-1-pyrroline-N-oxide (DMPO) and 2,2,6,6-tetramethyl-1-piperidinyloxy (TEMPO) were obtained from Aladdin Reagent Co., (China). 1,5-Diphenylcarbamide was purchased from Merck KGaA.

# Text S2. Catalyst synthesis and characterizations

The g-C_3_N_4_ nanosheet (CN-T) was synthesized based on our previous work^1^. Bulk carbon nitride (referred to as CN hereafter) was synthesized through the thermal decomposition of dicyandiamide. Simply put, dicyandiamide was placed in a semi-enclosed quartz boat in a static air environment and then calcined in a muffle furnace at 550°C for 4 h. Following that, the calcined material is ground, rinsed with ethanol and water, collected by centrifugation, and dried for a whole night at 80°C in an oven. Thermally exfoliated graphitic phase carbon nitride was prepared by secondary calcination of CN and named CN-T. The CN was dispersed in an open quartz boat and then calcined in a muffle furnace at 500°C for 4 h to acquire the final material named CN-T.

The surface chemical properties of the material and the changes in functional groups during the adsorption and degradation of phenol were characterized by Fourier Transform infrared spectroscopy (FTIR). The specific method is as follows: The catalyst samples at different reaction stages (pristine CN-T, CN-T after 30 min dark adsorption, CN-T after 30 min light irradiation and CN-T after 60 min light irradiation) were first collected by centrifugation at 3500 rpm for 5 min. The recovered catalysts were washed with ultrapure water three times to remove loosely adsorbed species, then dried in a vacuum oven at 60°C for 12 h. The dried samples were thoroughly ground and mixed with dry KBr powder at a mass ratio of about 1:100, and then pressed into transparent pellets under vacuum for FTIR testing. The infrared spectroscopy test was conducted using the Spectrum 400F spectrometer. The background scan was performed using a pure KBr pressure plate. All the spectra were collected at room temperature, and baseline correction and appropriate smoothing processing were performed using the software OMNIC that comes with the instrument.

Using the Shimadzu UV-3600Plus ultraviolet-visible-near-infrared spectrophotometer, equipped with an integrating sphere accessory, and with BaSO₄ as the reference, the diffuse reflectance spectra (DRS) of the materials before and after adsorbing phenol were tested. The scanning wavelength range for the test was set from 200 to 800 nm.

Free radical signals were detected using electron paramagnetic resonance (EPR) spectroscopy on a Bruker BioSpin GmbH EMX plus spectrometer. For spin-trapping, experiments employed 5,5-dimethyl-1-pyrroline-N-oxide (DMPO) and 2,2,6,6-tetramethylpiperidine-1-oxyl (TEMPO), with measurements conducted on a Bruker A300 accessory spectrometer.

The surface charge characteristics of the material were determined using a nanoparticle size analyzer (Zetasizer Pro). The test was conducted using disposable potential sample cells at a constant temperature of 25°C. Each sample was tested in parallel three times, and the results were averaged ± the standard deviation.

Transient photocurrent measurements of the photocatalysts were performed on a CHI660E electrochemical workstation using a standard three-electrode system. The as-prepared ITO glass coated with 4 mg of photocatalysts as the working electrode, platinum sheet as counter electrode, and the Ag/AgCl as reference electrode. Visible light LED lamps were adopted as the light source. The test was conducted in 0.1 M Na_2_SO_4_ electrolyte solution. In the control experiment, phenol was added to the solution.

The concentration of phenol was determined by high performance liquid chromatography (HPLC, Waters Alliance E2695, USA), which employed a C18 column (4.6 mm × 250 mm, 5 μm) and an ultraviolet-visible detector. The mobile phase used for detecting phenol was methanol/water (50:50, volume ratio), with a flow rate of 1.0 mL/min and a detection wavelength set at 270 nm.

# Text S3. Theoretical calculation.

In this study, density functional theory (DFT) was adopted to conduct theoretical calculations on the molecular interactions and electronic structure characteristics of the phenol/g-C_3_N_4_ complex system. All calculations were completed based on the Gaussian 16 package (Revision C.01), in which the optimization of the system’s geometric structure was carried out using the B3LYP-D3BJ functional with dispersion correction combined with the 6-31g(d) basis set^2–4^. The characteristics of π-π stacking interaction between molecules and the distribution law of frontier molecular orbitals were mainly studied, including the spatial distribution characteristics of the highest occupied molecular orbital (HOMO) and the lowest unoccupied molecular orbital (LUMO). The σ of pollutant molecules is taken from the values reported in the literature, while the electronic structure parameters such as E_HOMO_ are directly obtained through DFT calculation. The electron density distributions of four phenolic compounds were analyzed by the wave function of Multiwfn 3.8, and the visualization characterization of the three-dimensional electron cloud density was realized by using the VMD 1.9.3 program.

# Table S1 Basic Information on Substituted Phenols.

| Organic compound | Molecular formula | Structure | Molecular weight（g/mol） | Substituents |
| --- | --- | --- | --- | --- |
| phenol | C_6_H_6_O | 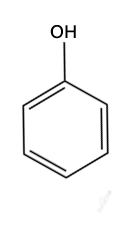 | 94.11 | - |
| 4-Chlorophenol | C_6_H_5_ClO | 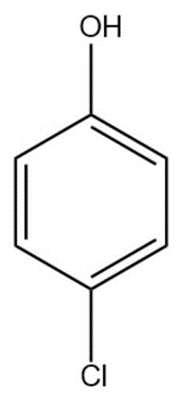 | 109.126 | -Cl |
| p-Nitrophenol | C_6_H_5_NO_3_ | 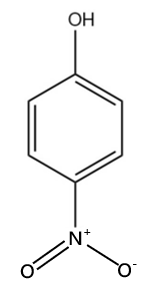 | 108.138 | -NO_2_ |
| 4-Methylphenol | C_7_H_8_O | 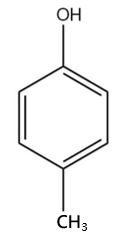 | 139.11 | -CH_3_ |

# Table S2. Comparison of photocatalytic removal of Phenol and Cr(VI) with recently reported catalysts.

| Catalysts | Dosage | Phenol/Cr(VI) (mg/L) | Reaction Conditions | 60 min Removal  (Phenol/Cr(VI)) | Ref. |
| --- | --- | --- | --- | --- | --- |
| BMO-400 | 1 g/L | 10&30 | 400 W halogen lamp | 42%&65% | 5 |
| CNM | 1 g/L | 20&10 | 250 W xenon lamp | 50%&60% | 6 |
| N-SSC/Bi_2_WO_6_ | 1 g/L | 10&10 | 300 W xenon lamp | 65%&25% | 7 |
| PANI@ZFCN | 1 g/L | 20&20 | 300 W xenon lamp | 80%&65% | 8 |
| HT-BVO | 1 g/L | 50&8 | 300 W xenon lamp | 82%&60% | 9 |
| Fe-Mn/AC | - | 100&30 | 50 mg/L O_3_ | 98%&92% | 10 |
| UiO-67-N,N-BA | 0.125 g/L | 20&50 | 300 W xenon lamp | 85%&100% | 11 |
| MEC-CeZnO | 0.5 g/L | 20&20 | UV lamp | 54%&42% | 12 |
| SnS_2_@CdS)/TiO_2_ | 1 g/L | 20&40 | 300 W xenon lamp | 65 %&85% | 13 |
| **CN-T** | **0.5** **g/L** | **10&5** | **30 W LED** | **99.6%&76.4%** | **This work** |

# Table S3. The relevant parameters for para-substitute organics degradation.

| Organic compound | σ^a^ | E_HOMO_^b^ (eV) | IP^c^ (eV) | E_1/2_^d^ (V) | *k* (×10**^-^**^2^ min**^-^**^1^) | R^2^ |
| --- | --- | --- | --- | --- | --- | --- |
| p-Nitrophenol | 0.78 | -6.922 | 9.10 | 0.924 | 4.11 ± 0.13 | 0.99 |
| 4-Chlorophenol | 0.23 | -6.094 | 8.69 | 0.653 | 10.35± 1.33 | 0.99 |
| phenol | 0 | -5.967 | 8.49 | 0.633 | 6.64± 0.58 | 0.99 |
| 4-Methylphenol | -0.17 | -5.745 | 8.30 | 0.543 | 35.10± 1.20 | 0.99 |

^a^σ values from ref 1^14^, ^b^obtained through theoretical calculations according to the reported density functional theory method, ^c^IP values from ref 2^15^, ^d^ E_1/2_ values from ref 3^16^. Same as below.


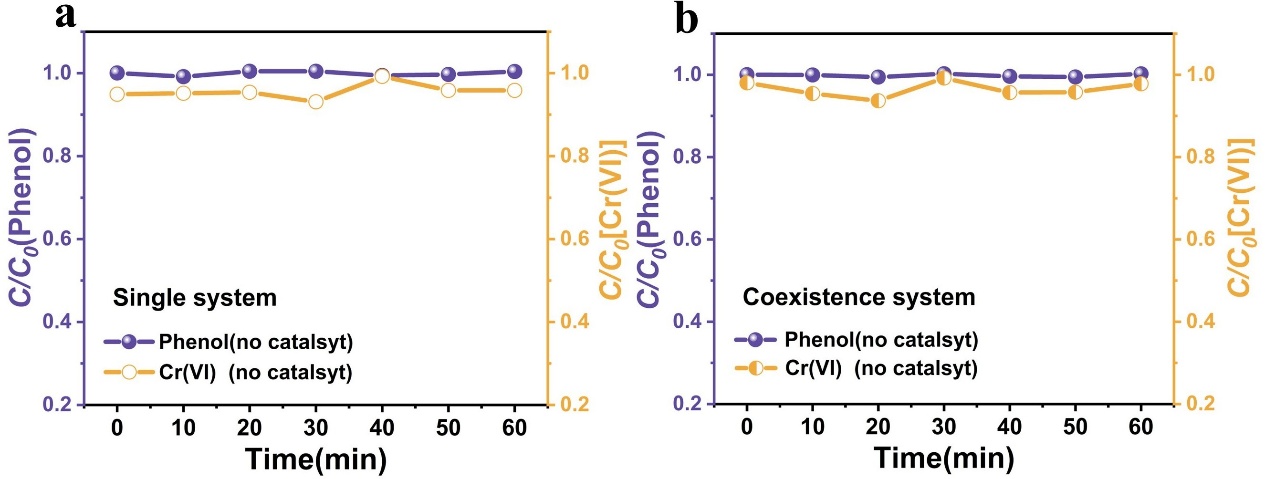


# **Fig. S1.** Removal of Phenol and Cr(Ⅵ) without CN-T catalysts in (a) single and (b) coexistence system.


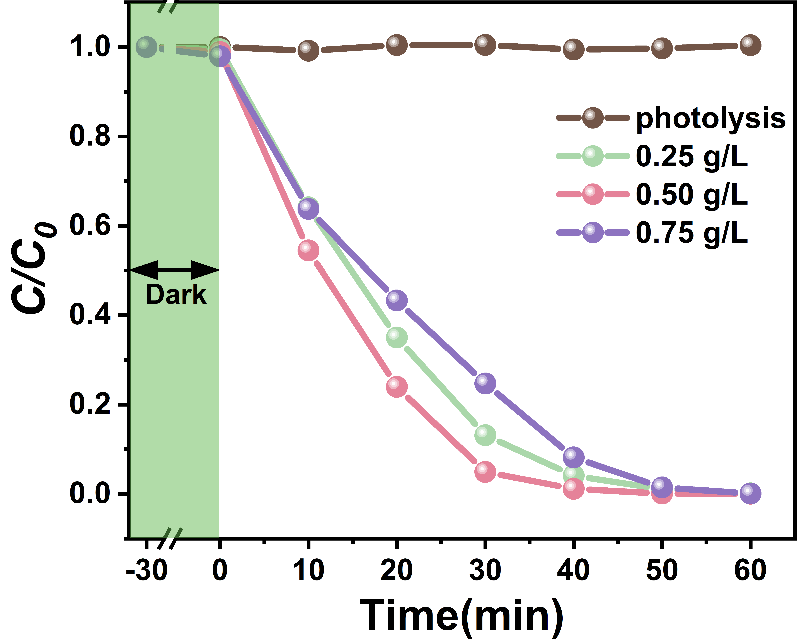


# Fig. S2. Comparison diagram of catalyst dosage for 10 mg/L phenol degradation.


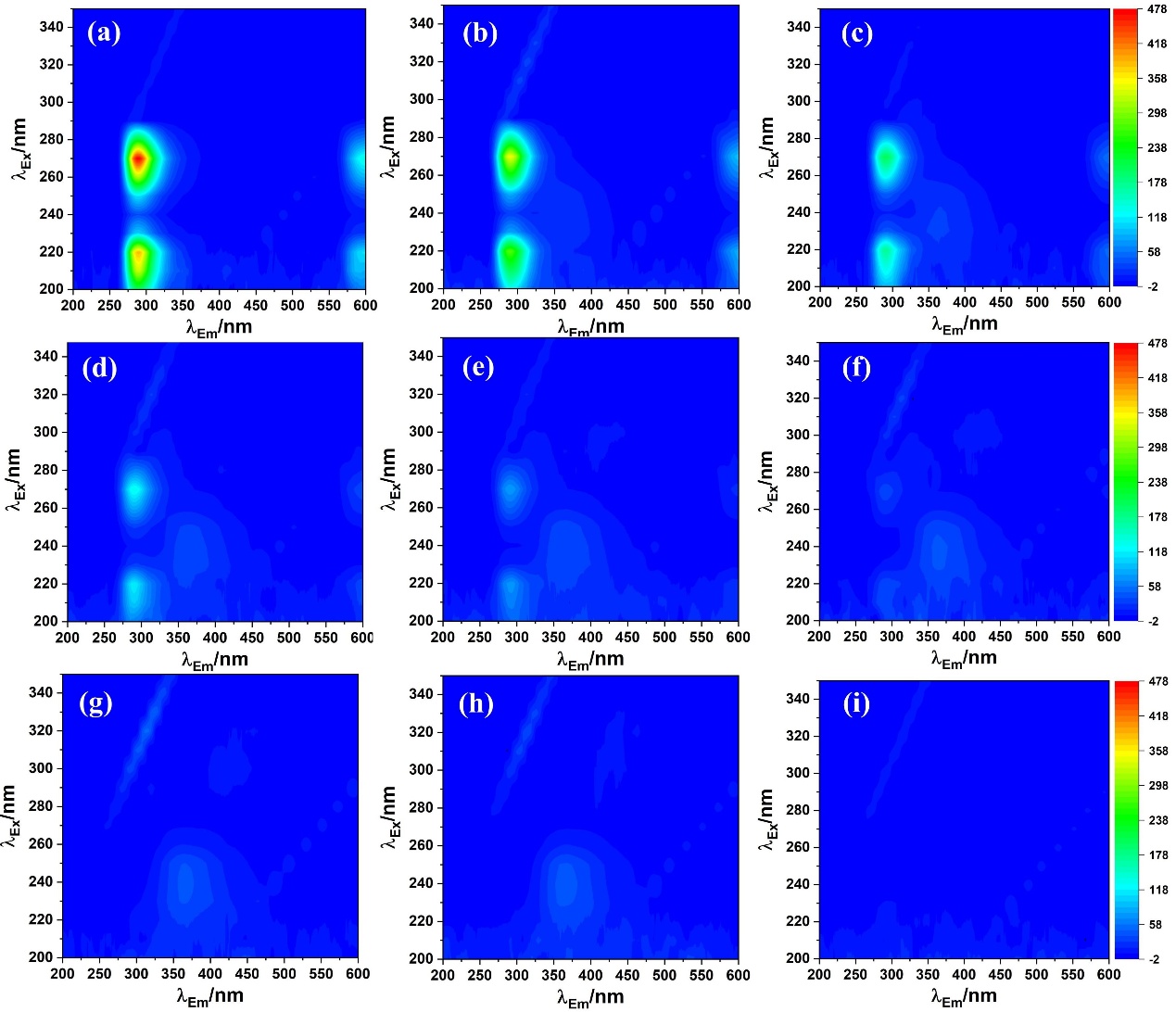


# Fig. S3. 3D EEMs of phenol in the coexistence system: (a) the original solution; (b-h) collected after irradiation times of 0, 10, 20, 30, 40, 50 and 60 min, respectively;(i) DI water.


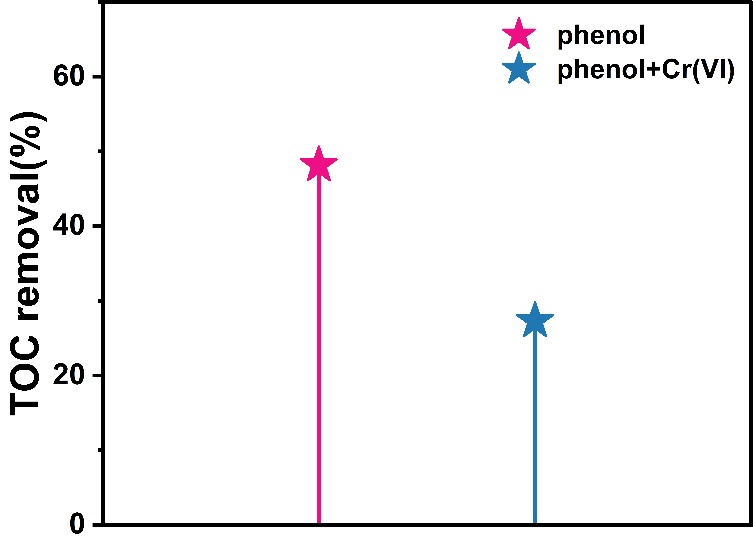


# Fig. S4. TOC removal efficiencies of phenol in single and coexistence systems after 60 min photocatalysis.


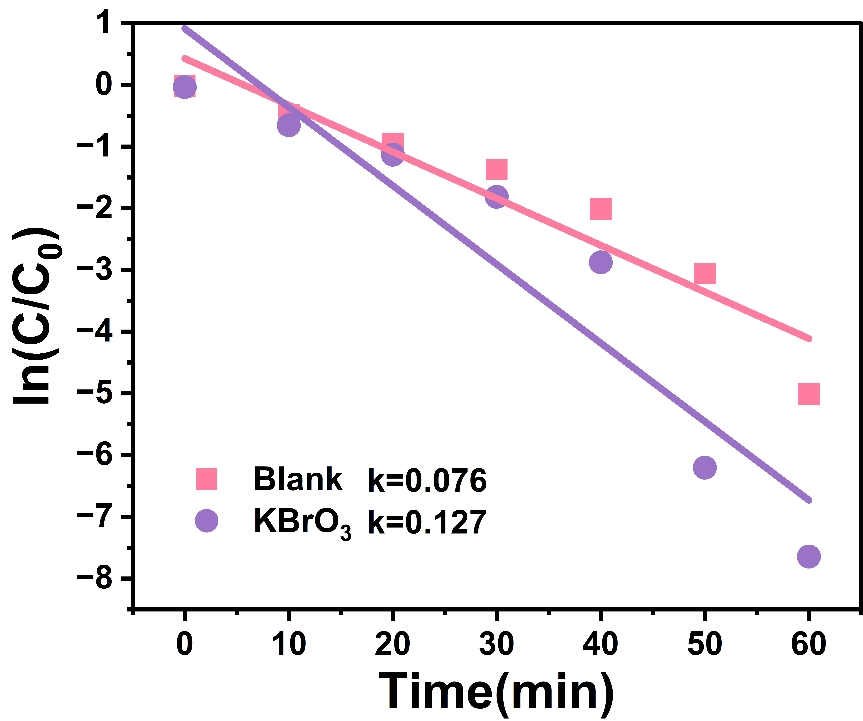


# Fig. S5. Comparison of phenol degradation rates with and without KBrO_3_ in coexistence system.


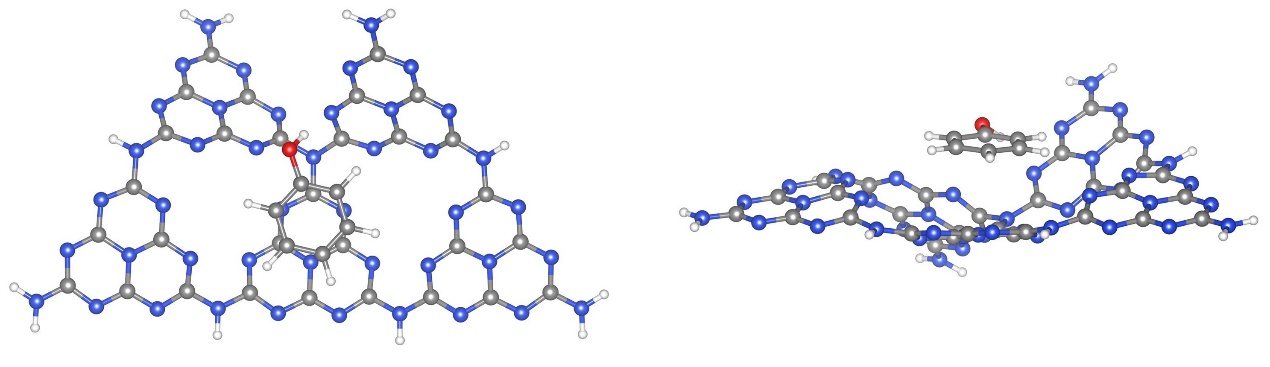


# Fig. S6. The optimized structure of Phenol/CN-T system (white, H; gray, C; blue, N; red, O).


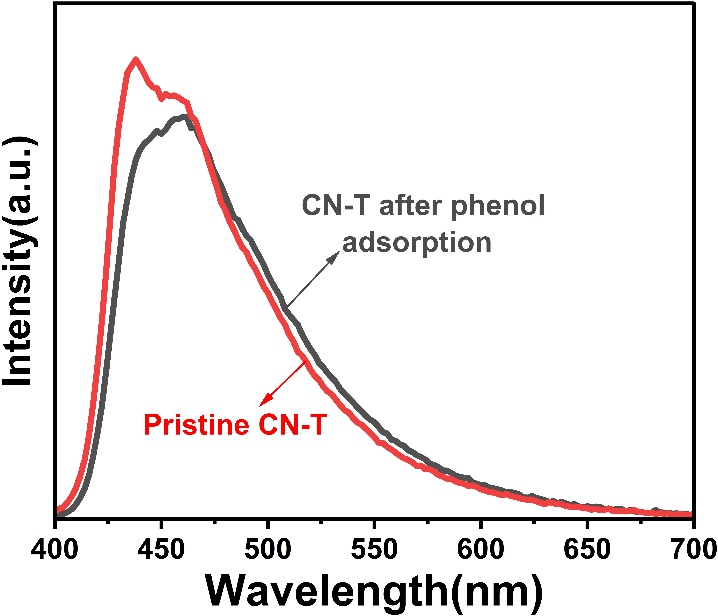


# Fig. S7. Dark-state PL spectra of CN-T before and after phenol adsorption.


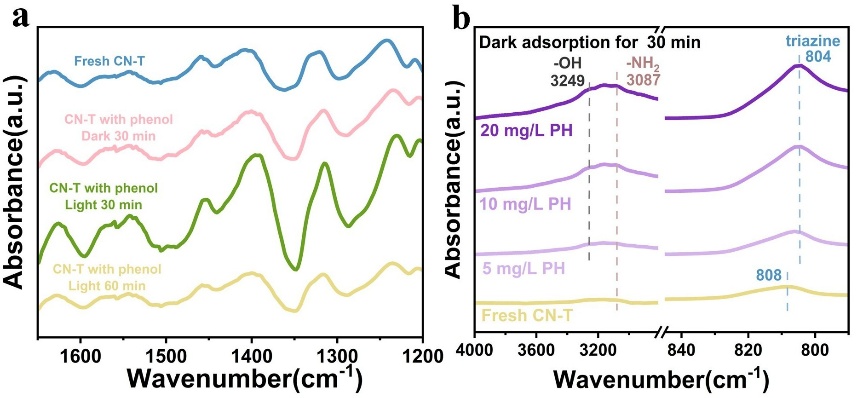


# Fig. S8. FTIR spectra of CN-T before and after adsorption of phenol and at different degradation time. (b) FTIR spectra after adsorption of varying phenol concentrations.


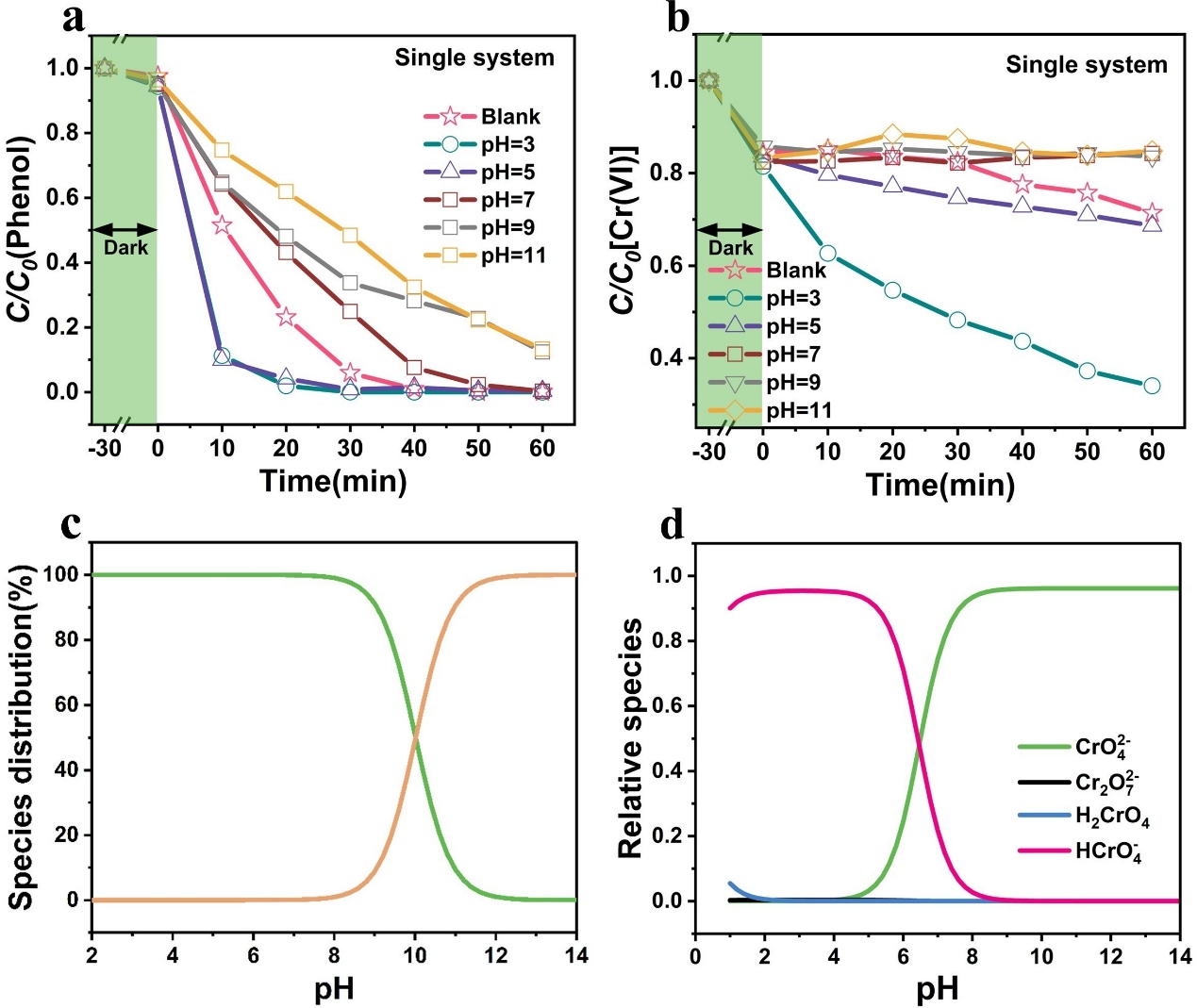


# Fig. S9. The influence of pH value on the removal of (a) phenol and (b) Cr(VI) in a single system. (c)Existence patterns of phenol in different pH solutions. (d) Cr(VI) speciation at different pH.


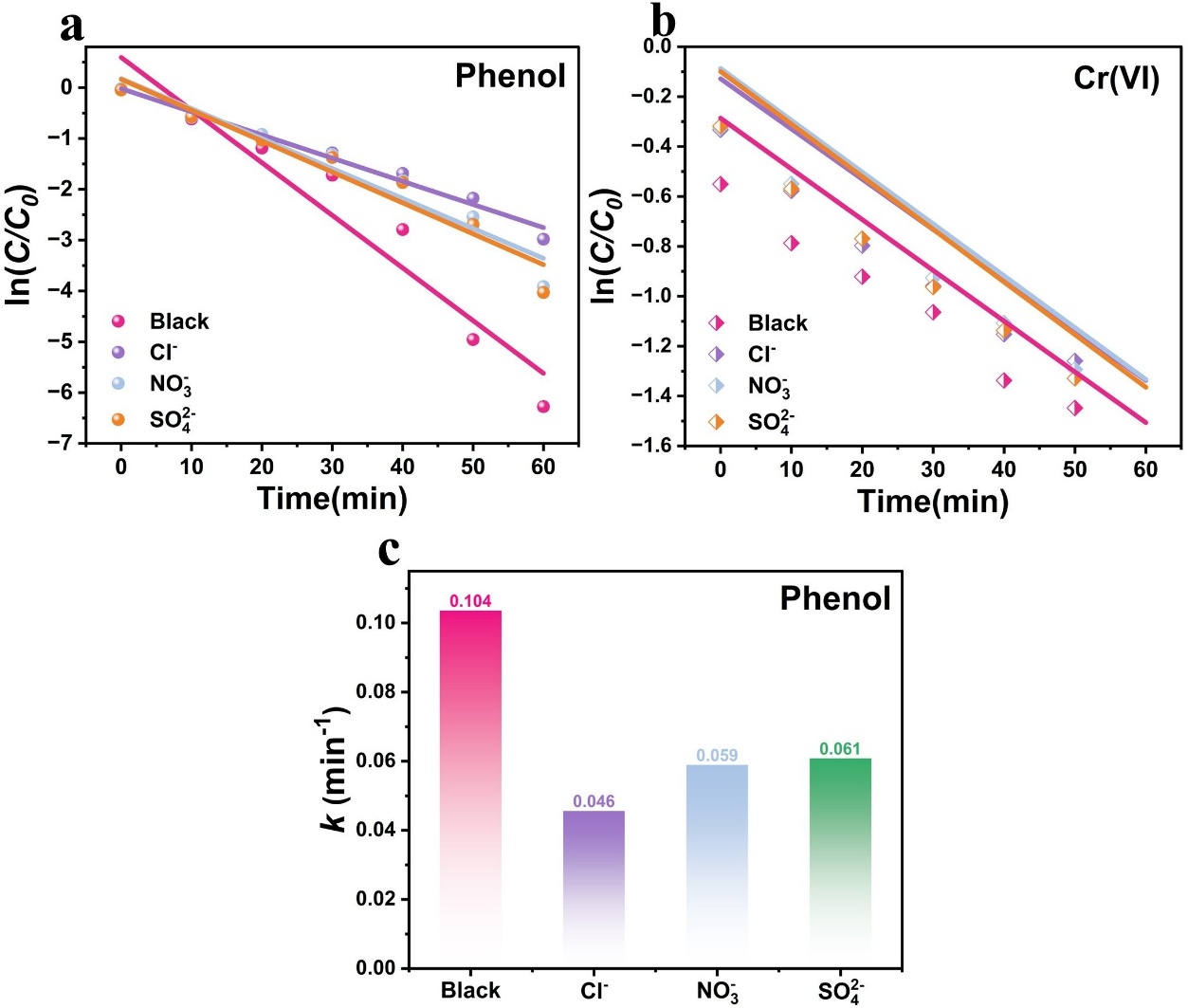


# Fig. S10. In the absence and presence of typical anions, a pseudo-first-order model was fitted for the (a) phenol degradation and (b) Cr(VI) reduction on CN-T. (c) Corresponding pseudo-first-order rate constants (k) of phenol degradation under different anion conditions.


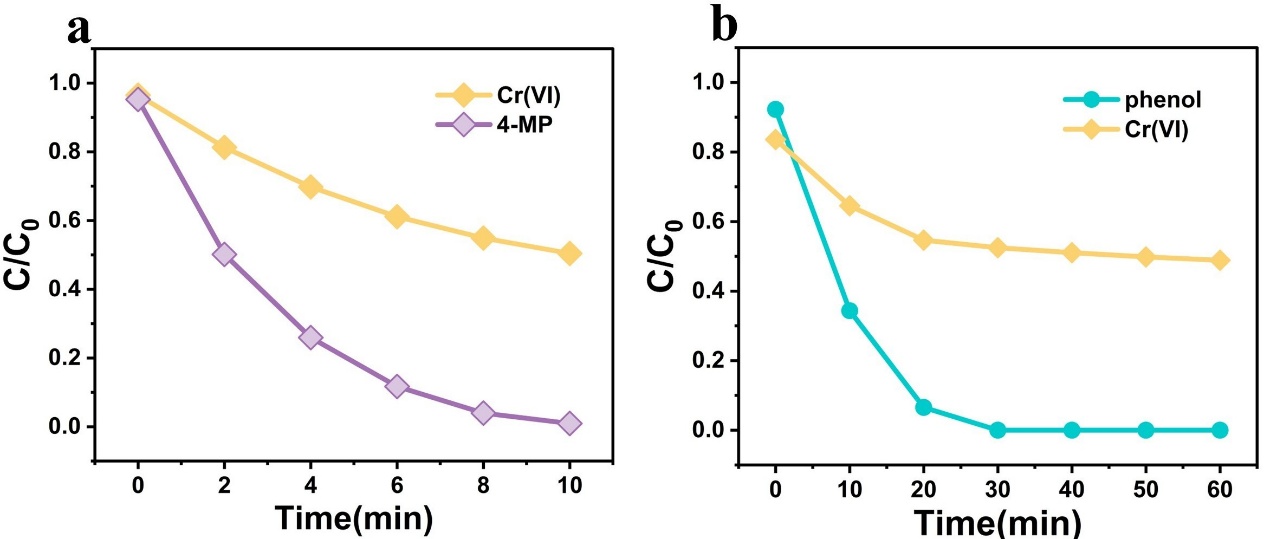


# Fig. S11. (a) Degradation activity of 4-MP and Cr(VI) in a coexisting system for 10 min. (b) Degradation performance of 5 mg/L phenol and Cr(VI) in a coexisting system.


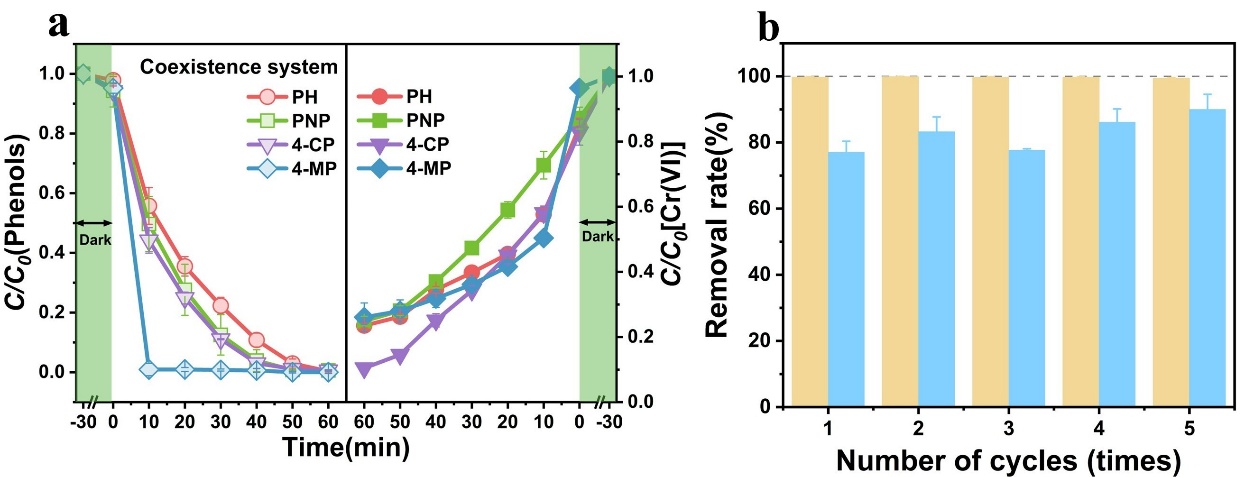


# Fig. S12. (a) Co-removal curves for substituted phenols and Cr(VI) in coexistence systems. (b) Final removal efficiencies of phenol and Cr(VI) over CN-T during 5 consecutive cyclic runs.


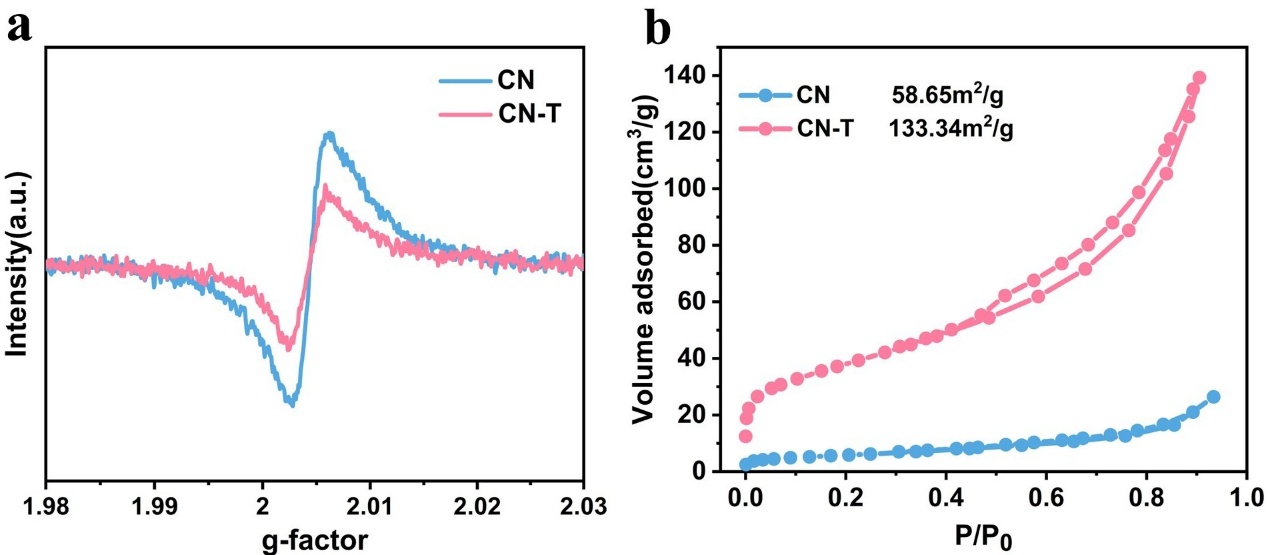


# Fig. S13. (a) ESR spectra. (b) N₂ adsorption-desorption isotherms.

The ESR spectra show that both CN and CN-T exhibit characteristic signals at g ≈ 2.003, but the signal intensity of CN-T is relatively weak, indicating that the thermal exfoliation process optimizes the defect structure of carbon nitride rather than simply increasing defects. Meanwhile, the BET results demonstrate that CN-T has a significantly higher specific surface area (133.34 m^2^/g) compared to pristine CN (58.65 m2/g), which means CN-T has a more porous structure. This structural advantage not only facilitates the mass transfer of phenol and Cr(VI) to the catalyst surface but also exposes more active sites for redox reactions—these structural changes directly explain why CN-T shows superior performance in the synergistic co-removal of phenol and Cr(VI) compared to pristine CN.

# References

1. Zhang, X.; Zhang, T.; Guo, H.; Wan, S.; Liu, X.; Sun, J.; Zhao, Y.; Dong, S.; Su, X.; Fan, M. Enhanced Generation and Effective Utilization of H_2_O_2_ for Simultaneous Aging of Plastics in the C_3_N_4_/Vis-LED System with the Addition of Phenol. *Chem. Eng. J.* **2024**, *500*, 157245. DOI: 10.1016/j.cej.2024.157245

2. Frisch, M. J.; Pople, J. A.; Binkley, J. S. Self‐consistent Molecular Orbital Methods 25. Supplementary Functions for Gaussian Basis Sets. *J. Chem. Phys.* **1984**, *80* (7), 3265–3269. DOI: 10.1063/1.447079

3. Stephens, P. J.; Devlin, F. J.; Chabalowski, C. F.; Frisch, M. J. Ab Initio Calculation of Vibrational Absorption and Circular Dichroism Spectra Using Density Functional Force Fields. *J. Phys. Chem.* **1994**, *98* (45), 11623–11627. DOI: 10.1021/j100096a001

4. Hariharan, P. C.; Pople, J. A. The Influence of Polarization Functions on Molecular Orbital Hydrogenation Energies. *Theor. Chim. Acta*. **1973**, *28* (3), 213–222. DOI: 10.1007/bf00533485

5. Shen, H.; Fu, F.; Xue, W.; Yang, X.; Ajmal, S.; Zhen, Y.; Guo, L.; Wang, D.; Chi, R. In Situ Fabrication of Bi2MoO6/Bi2MoO6-x Homojunction Photocatalyst for Simultaneous Photocatalytic Phenol Degradation and Cr(VI) Reduction. *J. Colloid Interface Sci.* **2021**, *599*, 741–751. DOI: 10.1016/j.jcis.2021.04.122

6. Patnaik, S.; Sahoo, D. P.; Parida, K. M. Bimetallic Co-Effect of Au-Pd Alloyed Nanoparticles on Mesoporous Silica Modified g-C3N4 for Single and Simultaneous Photocatalytic Oxidation of Phenol and Reduction of Hexavalent Chromium. *J. Colloid Interface Sci.* **2020**, *560*, 519–535. DOI: 10.1016/j.jcis.2019.09.041

7. He, Y.; Chen, Y.; Lei, S.; Zhong, J.; Li, M. Rich Oxygen Vacancies Facilitated Visible Light-Driven Removal of Phenol and Cr(VI) over Bi2WO6 Decorated by Sorghum Straw Carbon. *Colloids Surf., A*. **2022**, *641*, 128534. DOI: 10.1016/j.colsurfa.2022.128534

8. Patnaik, S.; Das, K. K.; Mohanty, A.; Parida, K. Enhanced Photo Catalytic Reduction of Cr (VI) over Polymer-Sensitized g-C3N4/ZnFe2O4 and Its Synergism with Phenol Oxidation under Visible Light Irradiation. *Catal. Today*. **2018**, *315*, 52–66. DOI: 10.1016/j.cattod.2018.04.008

9. Yu, T.; Lv, L.; Wang, H.; Tan, X. Enhanced Photocatalytic Treatment of Cr(VI) and Phenol by Monoclinic BiVO4 with {010}-Orientation Growth. *Mater. Res. Bull.* **2018**, *107*, 248–254. DOI: 10.1016/j.materresbull.2018.07.033

10. Zhang, J.; Shao, S.; Guo, Q.; Duan, X.; Liu, Y.; Jiao, W. Co-Removal of Phenol and Cr(VI) by High Gravity Coupled Heterogeneous Catalytic Ozonation-Adsorption. *Sep. Purif. Technol.* **2025**, *358*, 130297. DOI: 10.1016/j.seppur.2024.130297

11. Sun, Y.; Li, Z.; He, J.; Quan, K.; Qiu, H.; Chen, J. Regulated Zirconium-Based Metal Organic Frameworks via Ligand-Defect and Rare Earth Post-Coordination: A Synchronized Strategy for Enhancing Cr (VI) Adsorption and Phenol Photodegradation. *Chem. Eng. J.* **2026**, *533*, 174745. DOI: 10.1016/j.cej.2026.174745

12. Sheikh Hosseini Lori, M.; Delnavaz, M.; Khoshvaght, H. Synthesizing and Characterizing the Magnetic EDTA/Chitosan/CeZnO Nanocomposite for Simultaneous Treating of Chromium and Phenol in an Aqueous Solution. *Chin. J. Chem. Eng.* **2023**, *58*, 76–88. DOI: 10.1016/j.cjche.2022.05.010

13. Liu, Y.; Lin, Y.; Tang, J.; Liu, X.; Chen, L.; Tian, Y.; Fang, D.; Wang, J. Preparation of a Coated ZH-Scheme (SnS2@CdS)/TiO2(0 0 1) Photocatalyst for Phenol Degradation with Simultaneous Cr(VI) Conversion. *Appl. Surf. Sci.* **2022**, *574*, 151595. DOI: 10.1016/j.apsusc.2021.151595

14. Hansch, Corwin.; Leo, A.; Taft, R. W. A Survey of Hammett Substituent Constants and Resonance and Field Parameters. *Chem. Rev.* **1991**, *91* (2), 165–195. DOI: 10.1021/cr00002a004

15. Haynes, W. M.; Lide, D. R.; Bruno, T. J. CRC Handbook of Chemistry and Physics. *CRC Press*. **2016**

16. Zhan, C.-G.; Nichols, J. A.; Dixon, D. A. Ionization Potential, Electron Affinity, Electronegativity, Hardness, and Electron Excitation Energy:  Molecular Properties from Density Functional Theory Orbital Energies. *J. Phys. Chem. A*. **2003**, *107* (20), 4184–4195. DOI: 10.1021/jp0225774

1. Corresponding author. E-mail: dongsy@htu.edu.cn; (phone)86-13839079621. [↑](#footnote-ref-1)
2. Corresponding author. E-mail: xfsu@htu.edu.cn. [↑](#footnote-ref-2)
3. Corresponding author. E-mail: huchun@gzhu.edu.cn. [↑](#footnote-ref-3)
